# GPR116 alleviates acetaminophen-induced liver injury in mice by inhibiting endoplasmic reticulum stress

**DOI:** 10.1007/s00018-024-05313-0

**Published:** 2024-07-13

**Authors:** Qian Xiang, Na Li, Yan Zhang, Ting Wang, Ying Wang, Jinjun Bian

**Affiliations:** 1 Faculty of Anesthesiology, Changhai Hospital, Naval Medical University, 168 Changhai Road, Shanghai, 200433 China; 2 Department of Anesthesiology, Peking University Third Hospital, Beijing, 100191 China

**Keywords:** Drug-induced liver injury, Hepatocyte, GPCR, BiP, β-arrestin 1

## Abstract

**Background:**

Acetaminophen (APAP) overdose is a significant contributor to drug-induced liver injury worldwide. G-protein–coupled receptor 116 (GPR116) is an important homeostatic maintenance molecule in the body, but little is known about its role in APAP-induced liver injury (AILI).

**Methods:**

GPR116 expression was determined in both human and mouse AILI models. Hepatic function and damage response were analyzed in hepatocyte-specific *GPR116* deletion (*GPR116*^△HC^) mice undergoing APAP challenge. RNA-sequencing, immunofluorescence confocal, and co-immunoprecipitation (CO-IP) were employed to elucidate the impact and underlying mechanisms of GPR116 in AILI.

**Results:**

Intrahepatic GPR116 was upregulated in human and mice with AILI. *GPR116*^△HC^ mice were vulnerable to AILI compared to wild-type mice. Overexpression of GPR116 effectively mitigated AILI in wild-type mice and counteracted the heightened susceptibility of *GPR116*^△HC^ mice to APAP. Mechanistically, GPR116 inhibits the binding immunoglobulin protein (BiP), a critical regulator of ER function, through its interaction with β-arrestin1, thereby mitigating ER stress during the early stage of AILI. Additionally, the activation of GPR116 by ligand FNDC4 has been shown to confer a protective effect against early hepatotoxicity caused by APAP in murine model.

**Conclusions:**

Upregulation of GPR116 on hepatocytes inhibits ER stress by binding to β-arrestin1, protecting mice from APAP-induced hepatotoxicity. GPR116 may serve as a promising therapeutic target for AILI.

**Supplementary Information:**

The online version contains supplementary material available at 10.1007/s00018-024-05313-0.

## Introduction

Drug-induced liver injury is a common cause of acute liver failure and frequently leads to the withdrawal of drugs from the market. Acetaminophen (APAP) is a widely used antipyretic and analgesic. While generally safe at recommended doses, APAP can be toxic to the liver at high doses, leading to liver injury and failure [1]. In fact, APAP overdose is currently the leading cause of ALF in the United States, resulting in approximately 30,000 hospitalizations each year [2]. The development of strategies for the treatment of APAP-induced liver injury (AILI) has been extensive in recent years, but pharmacological options remain limited, providing an avenue for discovering new therapeutic targets.

In hepatocytes, APAP is converted by cytochrome P450 enzyme 2E1 (CYP2E1) to a toxic intermediate, N-acetyl-p-benzoquinone imine (NAPQI), which rapidly consumes glutathione (GSH) and binds to intracellular proteins to induce a series of signaling events, including endoplasmic reticulum (ER) stress [3–7]. ER stress has been shown to be closely associated with different stages of liver injury, and the death of hepatocytes triggered by ER stress may be crucial in drug-induced liver injury [8]. In the early stage of APAP hepatotoxicity, ER stress can be initiated when the influx of unfolded or misfolded proteins exceeds the folding capacity of the ER, triggering the terminal unfolded protein response (UPR) pathway, potentially leading to hepatocyte cell necrosis if excessively severe or prolonged [9]. However, the endogenous regulating mechanism responsible for alleviating ER stress in AILI remains largely unknown.

Adhesion G protein-coupled receptors (aGPCRs), the second largest GPCR class with exceptionally long ectodomains, are highly amenable to modulation by pharmaceuticals [10]. A wide variety of diseases have been linked to aGPCRs, which are involved in many physiological processes throughout the body [11]. Multiple aGPCRs are expressed in hepatocytes, mainly regulating fatty liver disease and diabetes [12]. G protein-coupled receptor 116 (GPR116), an aGPCR involved in tumorigenesis and cancer progression, is an important predictor of prognosis in cancers and is emerging as a promising therapeutic target [13–16]. Expressed in a variety of tissues, GPR116 plays a crucial role in regulating pulmonary surfactant homeostasis [17, 18], maintaining vascular endothelial junction integrity [19, 20], regulating kidney urine acid-base balance [21], and mediating insulin-sensitizing in systemic glucose homeostasis [22]. Despite our growing understanding of the biological roles of GPR116 in the lungs and other tissues, its function in the AILI remains unclear.

This study utilized hepatocyte-specific GPR116 knockout mice to establish an AILI model in order to investigate the specific role of GPR116 in AILI, elucidate its underlying mechanism, and identify a new intervention target for the treatment of AILI.

## Materials and methods

### Human samples

The human liver samples were obtained from patients with acute liver failure induced by APAP overdose who underwent liver transplantation (*n* = 5) in Renji Hospital of Shanghai Jiao Tong University (China), and healthy controls (HCs, *n* = 5) were obtained from liver donors. Liver tissues were collected during the transplantation procedure, and after fixation in formalin, embedded in paraffin for histological assessment. The informed consent was obtained from each subject. The study was carried out under the principles of the *Declaration of Helsinki* and approved by the research ethics boards of Renji Hospital. Demographic features of enrolled subjects are shown in Table 1.

**Table 1:** Demographic features of AILI patients and healthy controls (HCs) providing liver samples

	AILI (*n* = 5)	HCs (*n* = 5)
Age (years)	29 ± 7.9	33.2 ± 8.4
Gender (M/F)	3/2	2/3
ALT (U/L)	349 ± 313.8	34.5 ± 22.8
AST (U/L)	257.2 ± 173.4	17.4 ± 9.6
ALP (U/L)	152.4 ± 34.1	81 ± 30.2
γ-GT (U/L)	41.2 ± 12.8	27.8 ± 40.3
TBIL (µmol/L)	310.1 ± 79.3	12.5 ± 5.2
DBIL (µmol/L)	170.1 ± 57.7	2.1 ± 1.2
PT (s)	53.4 ± 22.5	10.5 ± 0.6
INR	5.0 ± 2.1	1.1 ± 0.13

*Abbreviations* M/F, Male and Female; ALT, Alanine aminotransferase; AST, Aspartate aminotransferase; ALP, Alkaline phosphatase; γ-GT, γ-glutamyl-transferase; TBIL, Total bilirubin; DBIL: Direct bilirubin; PT, Prothrombin time; INR, International normalized ratio

### Animal experiments

Male C57BL/6J mice (aged 8–12 weeks) were provided by the Experimental Animal Center of the Naval Military Medical University. Hepatocyte-specific GPR116 knockout (*GPR11*6^△HC^) mice were generated by crossing *GPR116*^flox/flox^ mice with *Alb*-Cre mice (B6.Cg- Speer6-ps1Tg(*Alb*-cre) 21Mgn/J, 003574) purchased from Jackson Laboratory as previously reported [23]. The above-mentioned mice were backcrossed with the C57BL/6J strain for nine generations, of which the healthy male knockout mice aged 8–12 weeks were used in the experiment. All knockout mice were constructed and identified by the Shanghai Model Organisms Center Inc. (Shanghai, China). All mice were housed in a specific pathogen-free environment at 23–25 °C and a 12/12 h light/dark cycle and were provided with water and chow ad libitum.

APAP (103-90-2, MCE, Monmouth Junction, NJ, USA) was dissolved in warm saline (0.9% NaCl; 55℃) and cooled to 37℃ before injection. To induce AILI, a single dose of 250 or 500 mg/kg body weight APAP was intraperitoneally (IP) injected in mice after overnight fasting (Fig. S1A) [24]. In ER stress inhibitor experiments, tauroursodeoxycholic acid (TUDCA; 14605-22-2, Selleck, Houston, TX, USA), dissolved in saline (250 mg/kg), was IP administered 3 times every 12 h along with APAP injection (Fig. S1B) [25]. In GPR116 ligand experiments, recombinant fibronectin type III domain containing 4 (FNDC4) (AG-40B-0124-C010, AdipoGen, Switzerland), dissolved in PBS (0.2 mg/kg), was IP administered 1 h after APAP injection (Fig. S1C) [22].

### Adenovirus and peptides

The mouse GPR116 gene (https://www.uniprot.org/uniprotkb?query =NM001081178.1& view=%20cards) was cloned into H340-pIRES2-EGFP-3xFLAG to generate GPR116-FLAG, and vectors were generated and packaged by Obio Technology (Shanghai, China). For ADV experiments, mice were intravenously injected with 10^9^ pfu of control (ADV-RAM) or GPR116-expressing (ADV-GPR116) ADVs 5 days before APAP treatment. GAP16-activating peptide was synthesized by GenScript Probio (Nanjing, China). Amino acid sequences for peptides: GAP16 (TSFSILMSPDSPDPGS), SCR (IFSDSTPSPDGLSMSP).

### Analysis of serum alanine aminotransferase (ALT) and aspartate aminotransferase (AST)

Serum ALT and AST levels were measured using ALT (MAK052, Sigma-Aldrich, St. Louis, MO, USA) and AST colorimetric assay kits (MAK055, Sigma-Aldrich), respectively, according to the manufacturer’s instructions.

### Western blotting and co-immunoprecipitation (CO-IP) analysis

For western blotting analysis, total protein was isolated from tissue or cell samples using RIPA lysis buffer (20–188, Sigma-Aldrich) with protease inhibitors. Samples were incubated for 10 min at 4 °C, vortexed, and centrifuged at 12 000 rpm for 10 min. Supernatants were collected and quantified for protein concentration using a BCA Protein Assay Kit (23,225, Thermo Fisher Scientific, Waltham, MA, USA). The protein samples were separated by 5–10% SDS-PAGE. Western blotting was performed as previously described, images were digitally captured using a ChemiScope 6 000 Exp (Clinx Science, Shanghai, China), and optical density was estimated using ImageJ software (National Institutes of Health, Bethesda, MD, USA). The antibodies used in this study are listed in Table 2.

**Table 2:** Antibodies used in the study

Antibody	Cat No.	Manufacturer	Concentration
GPR116	LS-C354382	LSBio	WB 1:1000; IHC 1:200; IF 1:300
anti-GPR116	ab111169	Abcam	0.4 µg/ml
CYP2E1	ab28146	Abcam	1:2000
CHOP	sc-7351	Santa Cruz	WB 1:2000; IHC 1:300
ATF4	11,815 S	CST	1:2000
ATF6	ab37149	Abcam	1:2000
p-eIF2α	3398 S	CST	1:1000
eIF2α	5324 S	CST	1:2000
sXBP1	12,782 S	CST	1:2000
tXBP1	ab37152	Abcam	1:2000
p-JNK	9251 S	CST	1:2000
JNK	9252 S	CST	1:2000
p-PERK	3179 S	CST	1:1000
PERK	3192 S	CST	1:2000
p-IRE1α	NB100-2323	Novus biologicals	1:2000
IRE1α	3294 S	CST	1:2000
Albumin	ab207327	Abcam	IF 1:500
F4/80	30,325 S	CST	IF 1:500
MPO	14,569 S	CST	IF 1:100
α-SMA	19245T	CST	IF 1:500
goat anti-mouse IgG-HRP	AT0098	Engibody Biotechnology	1:2000
goat anti-rabbit IgG-HRP	AT0097	Engibody Biotechnology	1:2000
GAPDH	5174T	CST	1:2000
β-Actin	3700 S	CST	1:2000

WB, western blotting; IHC, immunohistochemistry; IF, immunofluorescence

For CO-IP analysis, lysates that contain 500–1000 µg proteins were incubated with the primary antibody against GPR116, β-arrestin1 or IgG control (2 µg) overnight at 4 °C, followed by incubation with 25 µl of protein A/G magnetic beads. The beads were washed 5 times and eluted. The eluents were analyzed by western blotting.

### Quantitative real-time PCR (Q-PCR)

Total mRNA was extracted using a rapid extraction kit (220,010; Fastegen, Shanghai, China) according to the manufacturer’s instructions. cDNA was synthesized from 6.5 µg of mRNA using a PrimeScript RT-PCR Kit (RR014A, Takara Bio, Maebashi, Japan). mRNA levels were quantified by Q-PCR using TB Green Premix Ex Tap (RR420A, Takara Bio). Gene expression was normalized to that of the housekeeping gene *B2M* according to the 2^−ΔΔCt^ method. The primers used in this study are listed in Table 3.

**Table 3:** PCR primer sequences

Gene	Forward primer (5′-3′)	Reverse primer (5′-3′)
GPR116	ATGAGATCGCCAAGGACCTT	CCATGTATTCTTCCGCCACG
GPR110	CCAAGAGAAGCCAAACCTCC	TTCGATAAGCCAGCAGGATG
PDI	CAAGATCAAGCCCCACCTGAT	AGTTCGCCCCAACCAGTACTT
PERK	TAGGAAGATTCGAGCAGGGA	CTAGCCTCAGCAAGCCAGAG
ATF6	GAAGACTGGGAGTCGACGTT	ACTCCCAAGGCATCAAATCCAA
ATF4	CCTTCGACCAGTCGGGTTTG	CTGTCCCGGAAAAGGCATCC
CHOP	CGGAACCTGAGGAGAGAGTG	GTCTCCAAGGTGAAAGGCAG
sXBP1	GGTCTGCTGAGTCCGCAGCAGG	GGGGAAGGACATTTGAAACA
tXBP1	CTGAGCCCGGAGGAGAAA	TGCTCCAGCTCGCTCATC
CYP2E1	TGTGACTTTGGCCGACCTGTTC	CAACACACACGCGCTTTCCTGC
B2M	CGGCCTGTATGCTATCCAGA	GGGTGAATTCAGTGTGAGCC

### Histology and immunostaining analysis

Liver tissues were fixed in 10% formalin for 24 h before processing. Section (5-µm thick) from paraffin-embedded liver tissue were subjected to hematoxylin and eosin (H&E) staining, histological analysis, and various other types of staining. A standard H&E staining protocol was used. Sectioned slides were immunohistochemically stained for GPR116 and CHOP using previously described techniques [7, 14]. TdT-mediated dUTP nick-end labelling (TUNEL) was conducted using a fluorescein TUNEL Cell Apoptosis Detection Kit (G1501-50T, Servicebio, Wuhan, China) according to the manufacturer’s instructions. TUNEL and H&E staining signals were quantified using ImageJ software. The number of TUNEL^+^ cells was quantified in 10 random fields in each slide collected from per animal. Immunofluorescence (IF) staining was performed as described previously [26]. The cells were observed using a laser scanning confocal microscope (Leica, Mannheim, Germany).

### Cell isolation and treatment

Primary mouse hepatocytes were isolated from 8 weeks old mice as described previously [27], and were cultured in Williams’ medium E with 10% FBS without other supplements for 2 h to allow for attachment. AML-12 cells were cultured in DMEM/F-12 medium with 1% penicillin and streptomycin, 1% ITS (insulin-selenium-transferrin) and 10% FBS. AML-12 cells were transfected with control or GPR116-overexpressing (ov-GPR116) plasmid and incubated for 24 h. Cultured cells were incubated at 37 ℃ with 5% CO_2_. For APAP treatment, cells were treated in 12-well plates with APAP (10 mM) diluted in DMSO. For tunicamycin treatment, primary mouse hepatocytes were treated in 12-well plates with tunicamycin (10 µg/ml) diluted in DMSO.

### GSH levels

GSH levels were determined by using a GSH ELISA Kit (E-EL-0026c, Elabscience, Wuhan, China). One hundred milligrams of liver tissue from each mouse were taken and homogenized together with the lysis buffer from the kit. After centrifugation at 12 000×g for 10 min, the supernatant was collected, and the total concentration of GSH was measured according to the manufacturer’s instructions.

### CCK-8 assay

CCK-8 assay was carried out for detection of cell viability in AML-12 cells according to the manufacturer’s instructions. AML-12 cells were inoculated in 96-well plates with 2 × 10^3^ cells per well. Absorbance at 450 nm was recorded at specified time points using the CCK-8 kit, based on which the viability curve was plotted.

### APAP-cysteine protein adducts

APAP-Cysteine protein adducts were determined as previously described [28]. Briefly, liver tissues were homogenized and dialyzed to remove the free APAP-cysteine, then digested with protease, and the amount of APAP-cysteine was measured using high performance liquid chromatography-tandem mass spectrometry. Hepatic H_2_O_2_ levels were accessed using the Peroxidde Assay kit (Sigma-Aldrich, MAK311) according to the manufacturer’s protocol.

### Statistical analysis

Statistical analysis was performed using GraphPad Prism software (version 9.0; GraphPad Software, San Diego, CA, USA). Data were analyzed using two-tailed Student’s *t* test or one-way ANOVA followed by post hoc *t* tests. The results of survival experiments were analyzed using a Log-Rank test. Data are presented as the mean ± standard deviation (SD). Statistical significance was set at *P* < 0.05.

## Results

### Hepatic GPR116 expression is upregulated in human and mouse AILI

**Fig. 1:**
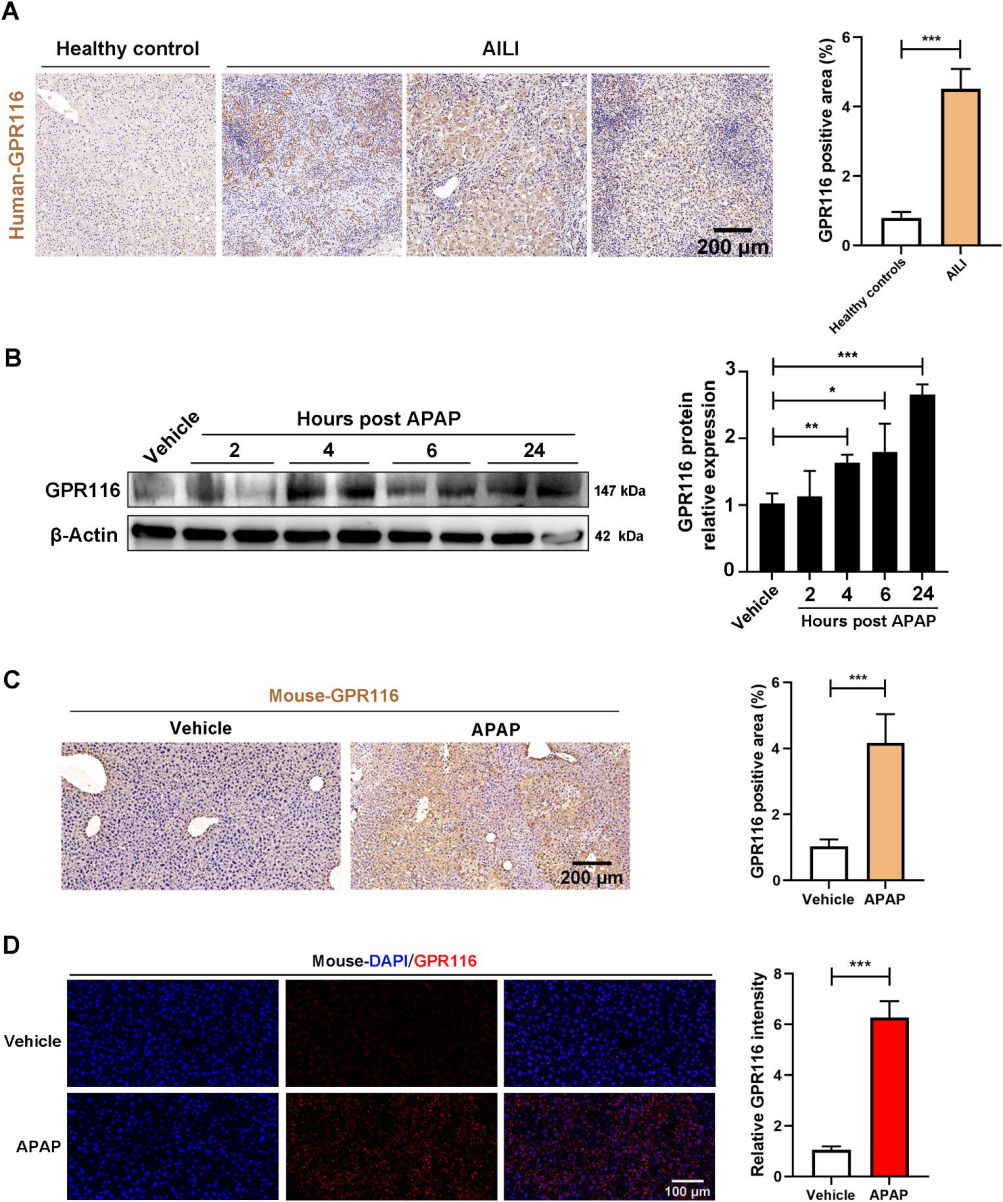
Hepatic expression of GPR116 is increased in human and mouse AILI. (**A**) Representative immunohistochemical (IHC) staining and quantification of GPR116 in livers of healthy controls and patients with AILI (*n* = 5 per group, scale bar: 200 μm). (**B**-**D**) C57BL/6J mice were injected with either APAP (250 mg/kg, IP) or an equal volume of saline (IP) as a vehicle control, and the livers of mice were harvested at different timepoints (*n* = 6 per group). (**B**) The protein level of GPR116 in the liver tissue homogenates was determined by western blotting and relative GPR116 level compared with β-Actin were shown as line graphs. (**C**) Representative IHC staining and quantification of GPR116 in liver tissues of mice treated with APAP for 4 h (scale bar: 200 μm). (**D**) Representative immunofluorescence (IF) staining of GPR116 (red) in liver tissues of mice treated with APAP for 4 h. Shown on the right are the quantifications of relative GPR116 signals. (scale bar: 100 μm). Statistical analysis was performed by Student’s *t*-test. Data are expressed as the mean ± SD. *P < 0.05; **P < 0.01; ***P < 0.001 as indicated. The experiments were repeated three times independently with similar results, and the data of one representative experiment was shown

To explore the potential involvement of GPR116 in AILI, we assessed the hepatic expression of GPR116 in both patients and mice with AILI. Immunohistochemistry (IHC) analysis revealed a significant increase in GPR116 expression in liver tissues from AILI patients compared to healthy controls (Fig. 1A). Additionally, protein expression of GPR116 (Fig. 1B) was found to be elevated in the livers of AILI mice. IHC and immunofluorescence (IF) analyses further confirmed the upregulation of GPR116 in parenchymal cells (hepatocytes) of AILI mice (Fig. 1C, D). Moreover, IF analysis revealed that GPR116 did not exhibit an increased expression in non-parenchymal cells, including macrophages (F4/80), neutrophils (myeloperoxidase, MPO), and hepatic stellate cells (α-smooth muscle actin, α-SMA) following APAP challenge (Fig. S1D-F). These findings collectively suggest a potential involvement of hepatocyte GPR116 in the pathogenesis of AILI.

### Hepatocyte-specific knockout of *GPR116* exacerbates AILI in mice

**Fig. 2:**
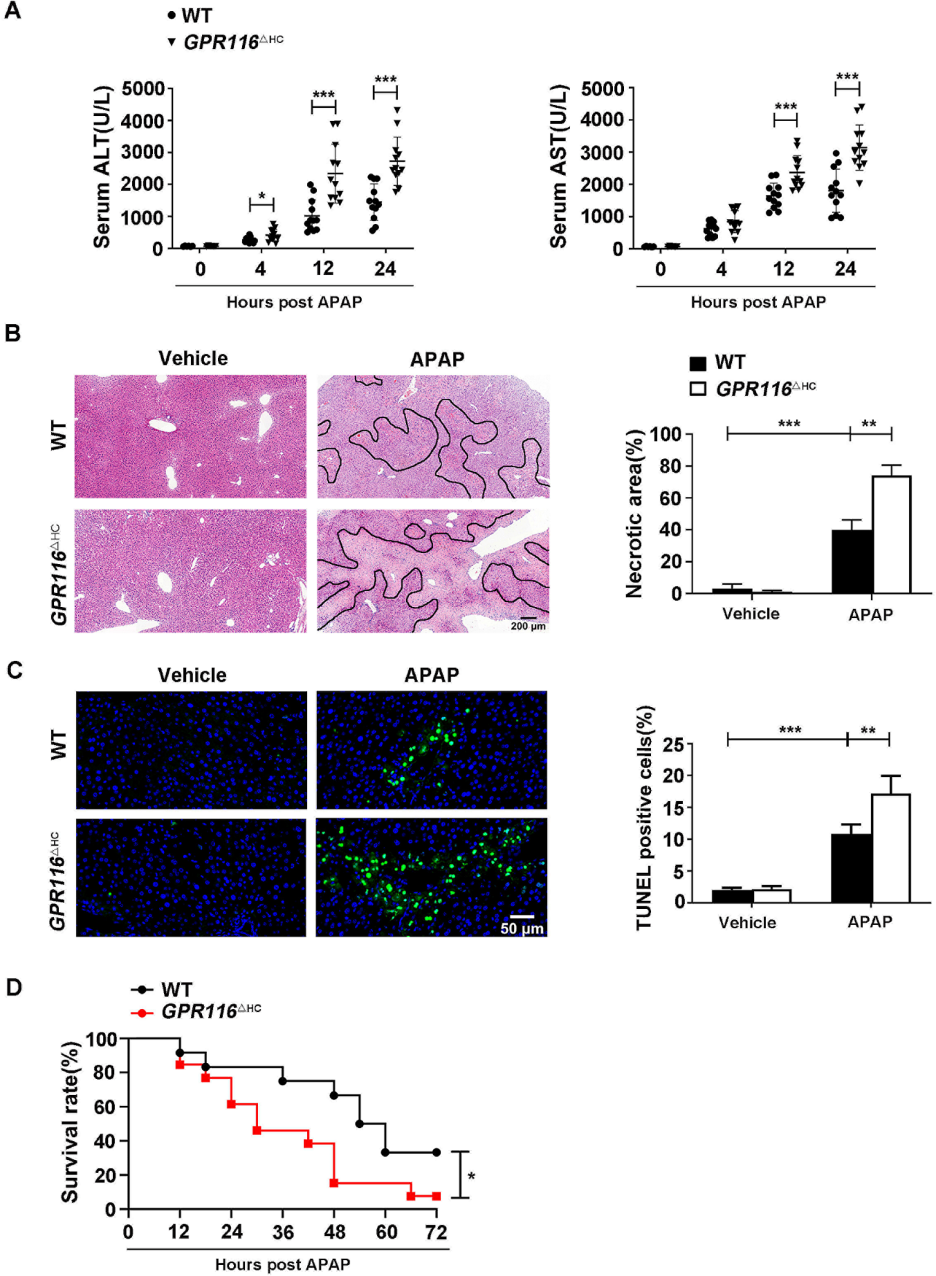
Hepatocyte-specific knockout of GPR116 exacerbates AILI. (**A**-**C**) WT and *GPR116*^△HC^ mice were injected with either APAP (250 mg/kg, IP) or an equal volume of saline (IP) as a vehicle control (*n* = 6–12 per group). (**A**) Serum levels of ALT and AST in mice measured at different timepoints. (**B**) Left, representative H&E staining (scale bar: 200 μm) of liver tissues from mice harvested at 24 h post-APAP injection; right, quantification of necrotic areas by ImageJ software (*n* = 3 per group). (**C**) Left, representative TUNEL staining (scale bar: 50 μm) of liver tissues from mice harvested at 24 h post-APAP injection; right, quantification of the number of TUNEL-positive cells per field by ImageJ software (*n* = 3 per group). (**D**) The survival rate of WT (*n* = 11) and *GPR116*^△HC^ (*n* = 12) mice for 72 h after injection with a lethal dose of APAP (500 mg/kg, IP). Statistical analysis was performed by Student’s *t*-test and one-way ANOVA. The survival rates were analyzed by the Log-Rank test. Data are expressed as the mean ± SD. *P < 0.05; **P < 0.01; ***P < 0.001 as indicated. Data are pooled from at least two independent experiments

In order to examine the functional significance of hepatocyte GPR116 in AILI, we created *GPR116* hepatocyte-specific knockout (*GPR116*^△HC^) mice, as outlined in a previous publication [23]. The efficacy of *GPR116* knockout at the hepatic protein level was validated through western blot analysis, with no discernible knockout observed in the lung, kidney, or heart tissues (Fig. S2A). The *GPR116*^△HC^ mice exhibited normal health and fertility under basal conditions, displaying no overt phenotypic abnormalities in terms of gross organ appearance or weight (Fig. S2B, C). In comparison to *GPR116*^Hep+/+^ (wildtype, WT) mice, the absence of GPR116 exacerbated the elevation of serum ALT and AST levels following APAP administration in a time-dependent manner (Fig. 2A). Additionally, *GPR116*^△HC^ mice displayed increased levels of liver necrosis as determined by histological analysis (Fig. 2B) and TUNEL staining (Fig. 2C). Furthermore, *GPR116*^△HC^ mice exhibited a significantly higher mortality rate compared to WT mice when administered a lethal dose of APAP (Fig. 2D). Together, these findings suggest that GPR116 may play a protective role in the context of AILI.

### Hepatocyte-specific knockout of *GPR116* aggravates ER stress in APAP-challenged mice

**Fig. 3:**
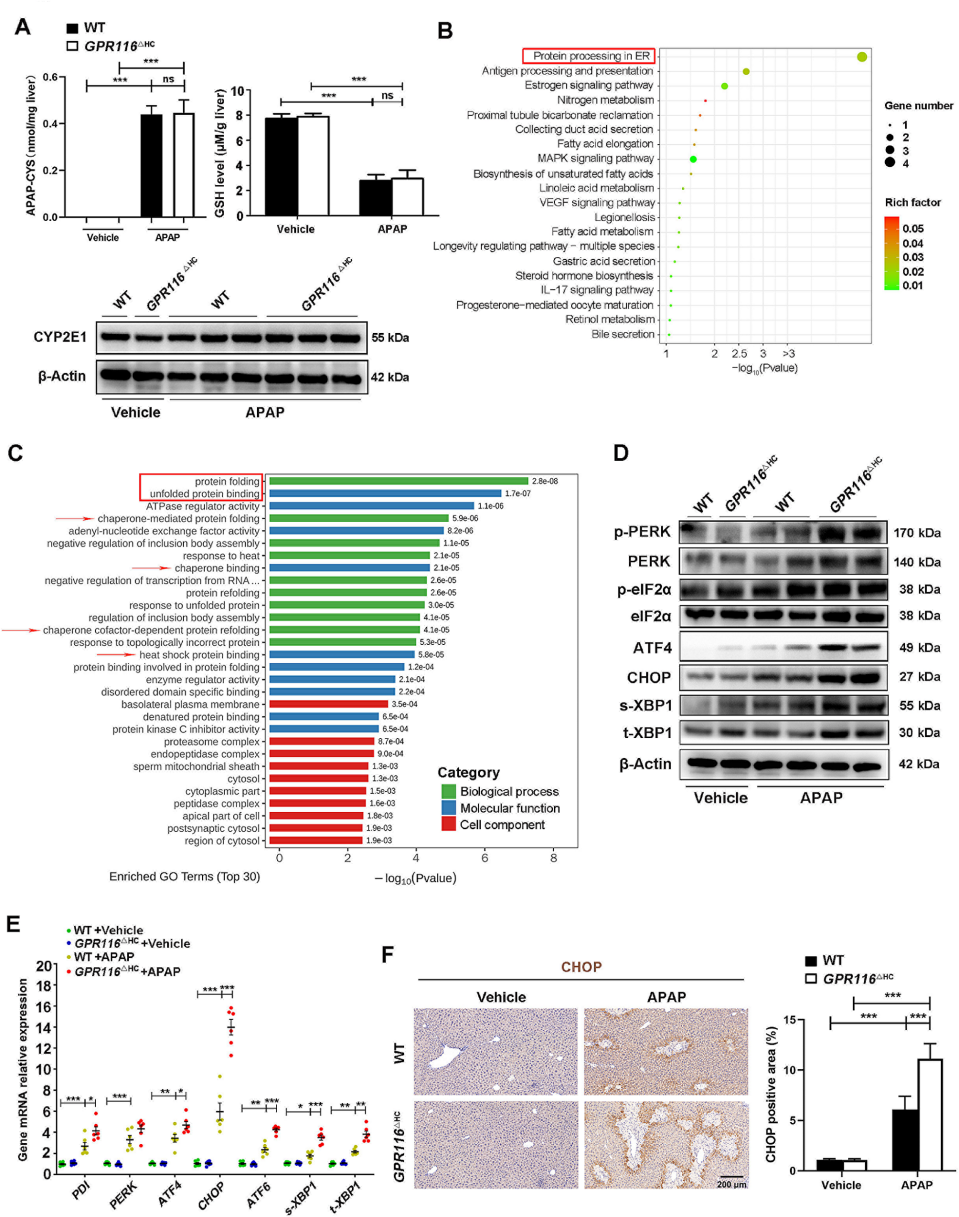
Hepatocyte-specific knockout of *GPR116* aggravates APAP-induced ER stress. (**A**-**F**) WT and *GPR116*^△HC^ mice were treated with either APAP (250 mg/kg, IP) or an equal volume of saline (IP) as a vehicle control (*n* = 4–8 per group) for 4 h. (**A**) The APAP-CYS level, CYP2E1 protein level and GSH level in the liver tissue homogenates of mice. (**B**) KEGG analysis showed the top 20 enriched pathways in the *GPR116*^△HC^ group versus WT controls (*n* = 3 per group). (**C**) GO functional enrichment analysis of RNA-seq data. (**D**) Western blotting of ER stress markers in liver tissue homogenates of mice. (**E**) mRNA levels of ER stress markers in liver tissue homogenates of mice. (**F**) Representative IHC staining and quantification of CHOP in liver tissues of mice (scale bar: 200 μm). Statistical analysis was performed by one-way ANOVA. Data are expressed as the mean ± SD. *P < 0.05; **P < 0.01; ***P < 0.001 as indicated. Data of A, D-F are pooled from at least two independent experiments

We further conducted a detailed investigation into the intrahepatic signaling pathways of GPR116 in the context of AILI. Our findings suggested that GPR116 did not play a significant role in APAP metabolism, as evidenced by no significant difference in APAP-Cysteine protein adducts, glutathione (GSH) consumption, and CYP2E1 expression observed in *GPR116*^△HC^ and wild-type (WT) mice following APAP overdose (Fig. 3A, Fig. S3A, B).

Since GPR116 began to be upregulated significantly after 4 h APAP treatment, and there were already differences in ALT and AST levels of *GPR116*^△HC^ and WT mice at this timepoint, liver tissues harvested from two groups of mice treated with APAP for 4 h were subjected to RNA-seq analysis. The results revealed 24 differentially expressed genes, of which 20 genes were upregulated following GPR116 deletion (Fig. S4A). Kyoto Encyclopedia of Genes and Genomes (KEGG) pathway enrichment analysis showed that the interacting proteins related to the dominant genes were enriched in ER protein processing-related signaling pathways (Fig. 3B). Additionally, Gene Ontology (GO) enrichment analysis showed that GPR116 deficiency upregulated protein folding and unfolded protein binding (Fig. 3C). An overload of ER stress is associated with APAP hepatotoxicity and multiple molecules, including CHOP, play vital roles in AILI. GPR116 deletion markedly exacerbated the increase in ER stress markers at the protein (e.g., PERK-eIF2α-ATF4-CHOP, s-XBP1, and t-XBP1) and mRNA (e.g., PDI, PERK, ATF4, CHOP, ATF6, s-XBP1, and t-XBP1) levels in the livers of mice 4 h post APAP injection (Fig. 3D, E), indicating that different branches of the ER stress pathway are involved. IHC analysis showed that CHOP expression was increased in the livers of APAP-treated *GPR116*^△HC^ mice compared with WT mice (Fig. 3F). In summary, the intolerance of APAP challenge exhibited by *GPR116*^△HC^ mice may be originated from uncontrolled ER stress.

### Targeting ER stress rescues *GPR116* deletion-induced deterioration of APAP-induced hepatotoxicity

**Fig. 4:**
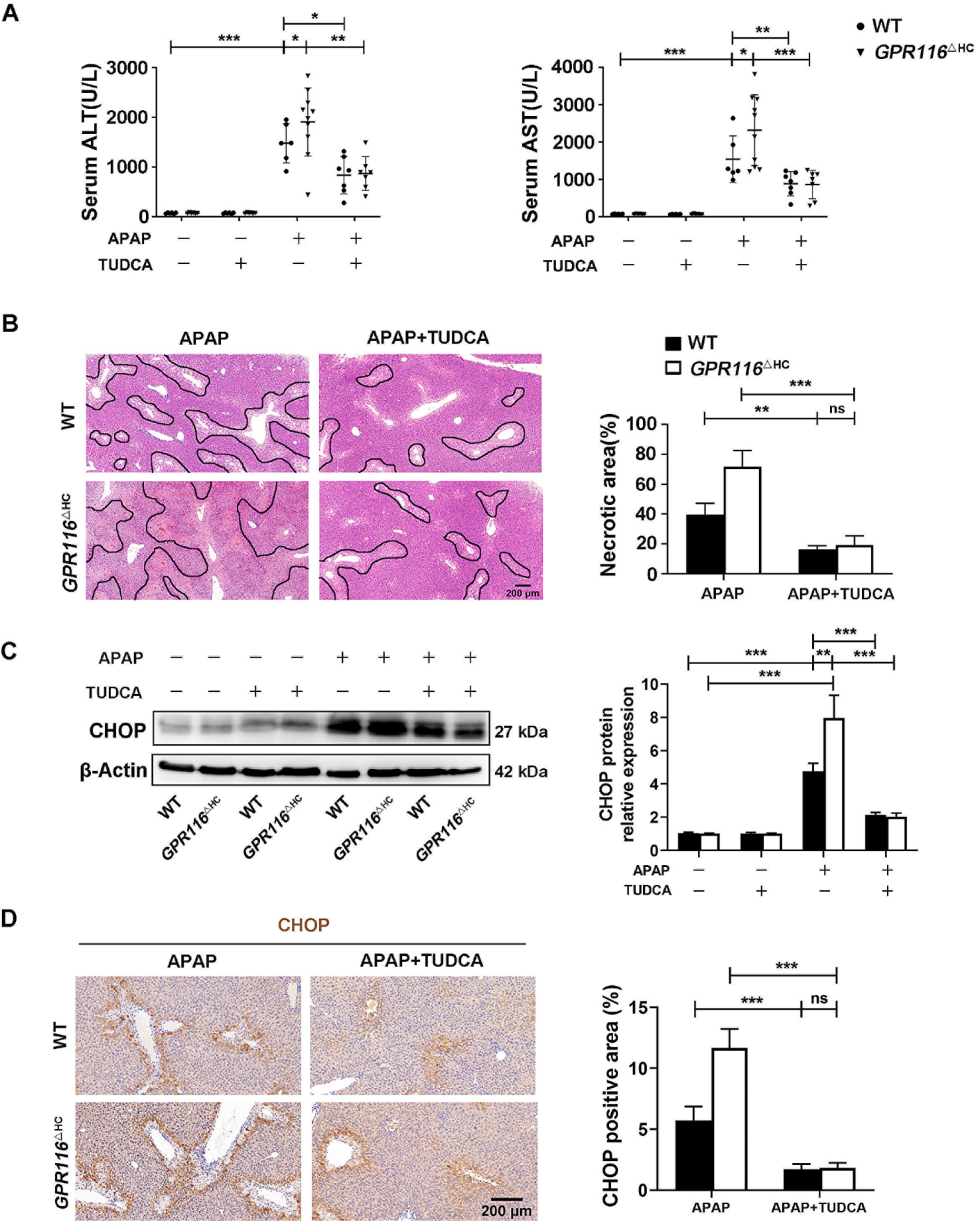
Targeting ER stress rescues *GPR116* deletion-induced deterioration of APAP-induced hepatotoxicity. (**A**) Serum levels of ALT and AST in mice. (**B**) Representative H&E staining of liver tissues from mice (scale bar: 200 μm) and quantification of necrotic areas (*n* = 3 per group). (**C**) Western blotting of CHOP in liver tissue homogenates of mice and relative CHOP level compared with β-Actin were shown as line graphs. (**D**) Representative IHC staining and quantification of CHOP in liver tissues of mice (scale bar: 200 μm). Statistical analysis was performed by one-way ANOVA. Data are expressed as the mean ± SD. *P < 0.05; **P < 0.01; ***P < 0.001; no significance (ns) *P* > 0.05 as indicated. Data are pooled from at least two independent experiments

To determine the role of ER stress in GPR116 signaling following APAP challenge, in vivo rescue assays were performed (Fig. 4A). Treatment with TUDCA (an ER stress antagonist, Fig. S4B-D) resulted in a decrease in serum ALT and AST, as well as a reduction in liver tissue necrosis, in WT mice 24 h post APAP injection. Notably, TUDCA reversed the disease phenotype of *GPR116*^△HC^ APAP mice effectively (Fig. 4B, C). Consistent with these findings, western blot and IHC staining demonstrated that TUDCA attenuated the expression of ER stress marker-CHOP in liver tissues of both *GPR116*^△HC^ and WT mice (Fig. 4D, E). Consequently, it can be concluded that GPR116 acts as a protective factor against APAP-induced hepatotoxicity by impeding ER stress.

### Re-expression of GPR116 in mice alleviates APAP-induced hepatotoxicity

**Fig. 5:**
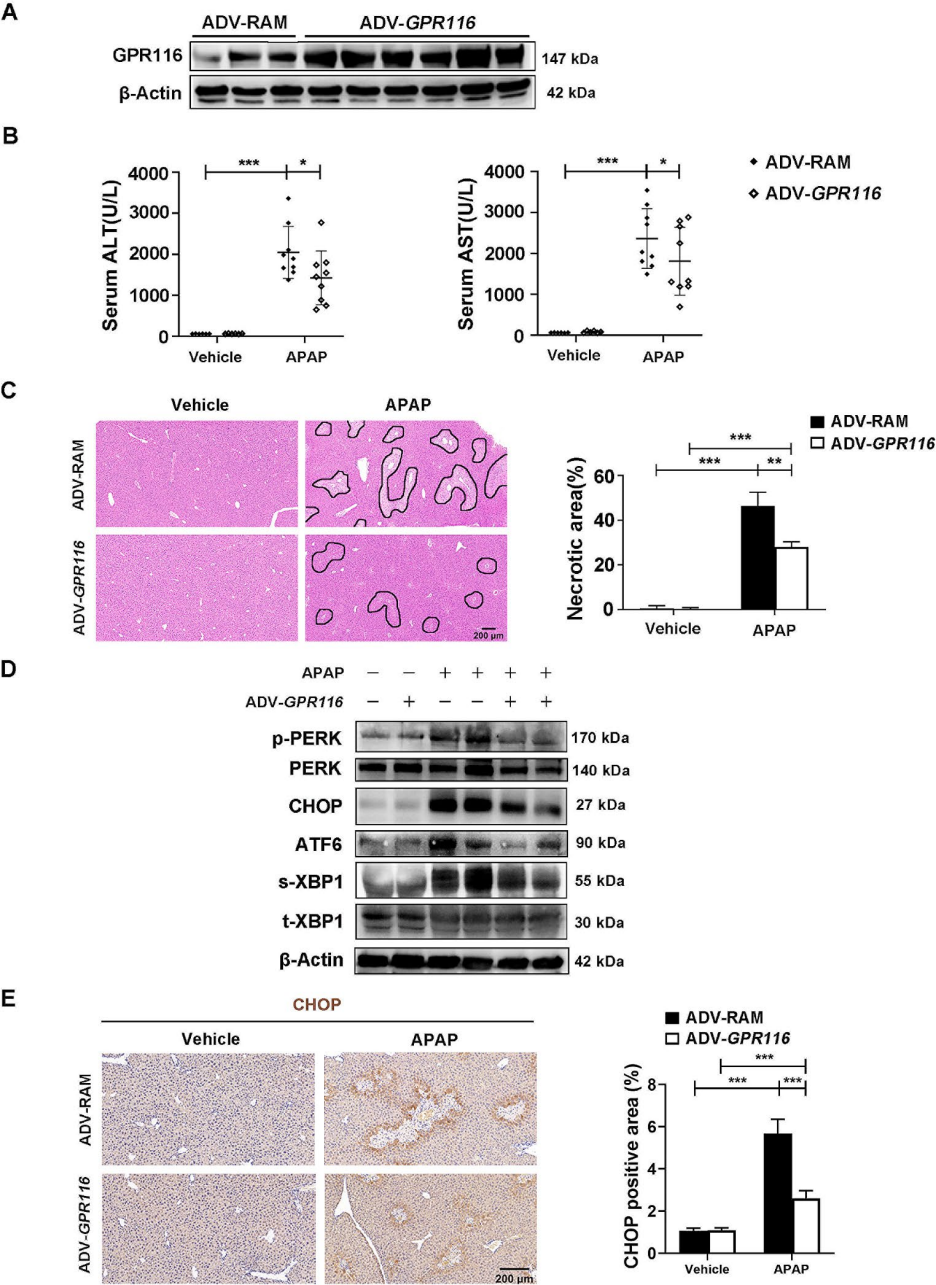
Re-expression of GPR116 alleviates APAP-induced hepatotoxicity. (**A**-**E**) C57BL/6J mice were injected with control (ADV-RAM) or GPR116-expressing (ADV-*GPR116*) adenovirus 5 days before APAP treatment (250 mg/kg, IP) (*n* = 9 per group). (**A**) The protein level of GPR116 in the liver tissue homogenates was determined by western blotting. (**B**) Serum levels of ALT and AST in mice 24 h after APAP injection. (**C**) Representative H&E staining of liver tissues from mice (scale bar: 200 μm) and quantification of necrotic areas 24 h after APAP injection (*n* = 3 per group). (**D**) Western blotting of ER stress markers in liver tissue homogenates of mice 4 h after APAP injection. (**E**) Representative IHC staining and quantification of CHOP in liver tissues of mice 4 h after APAP injection (scale bar: 200 μm). Statistical analysis was performed by one-way ANOVA. Data are expressed as the mean ± SD. *P < 0.05; **P < 0.01; ***P < 0.001 as indicated. Data are pooled from at least two independent experiments

**Fig. 6:**
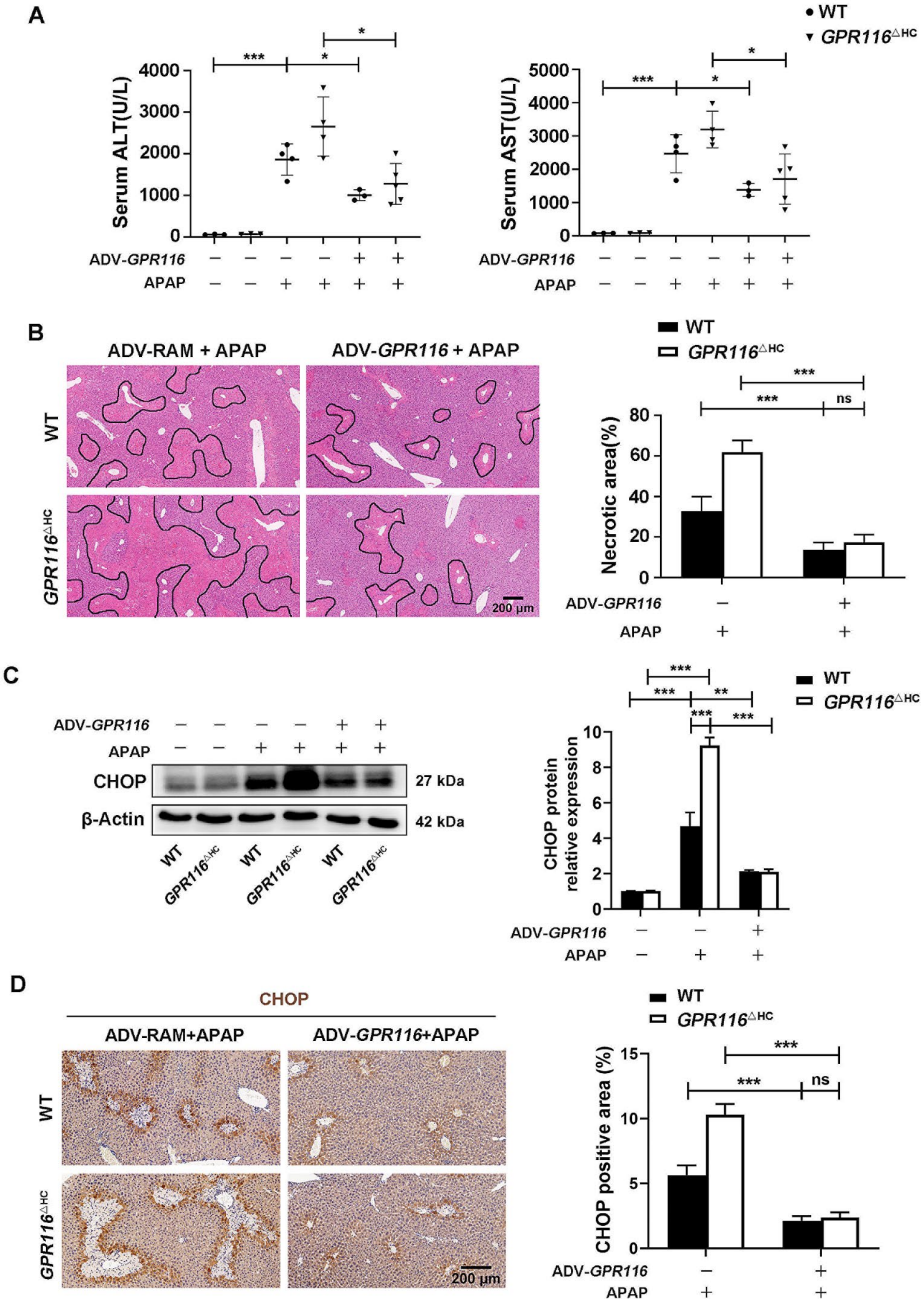
Re-expression of GPR116 in *GPR116*^△HC^ mice reverse exacerbation of APAP-induced hepatotoxicity. (**A**-**E**) WT and *GPR116*^△HC^ mice were injected with control (ADV-RAM) or GPR116-expressing (ADV-*GPR116*) adenovirus 5 days before APAP treatment (250 mg/kg, IP) (*n* = 3–9 per group). (**A**) Serum levels of ALT and AST in mice 24 h after APAP injection. (**B**) Representative H&E staining of liver tissues from mice (scale bar: 200 μm) and quantification of necrotic areas 24 h after APAP injection (*n* = 3 per group). (**C**) Western blotting of CHOP in liver tissue homogenates of mice 4 h after APAP injection and relative CHOP level compared with β-Actin were shown as line graphs. (**D**) Representative IHC staining and quantification of CHOP in liver tissues of mice 4 h after APAP injection (scale bar: 200 μm). Statistical analysis was performed by one-way ANOVA. Data are expressed as the mean ± SD. *P < 0.05; **P < 0.01; ***P < 0.001 as indicated. Data are pooled from at least two independent experiments

In order to ascertain the hepatoprotective role of GPR116 in vivo, we conducted an assessment on the impact of GPR116 overexpression on APAP-induced hepatotoxicity. C57BL/6J mice were administered a GPR116-expressing adenovirus (ADV-*GPR116*) or control virus (ADV-RAM) prior to APAP administration (Fig. 5A, Fig. S5A-C). It was worth noting that GPR116 overexpression provided protection against AILI in the mice, as evidenced by the reduction in serum ALT and AST levels (Fig. 5B), and the mitigation of pathological liver injury (Fig. 5C). Moreover, GPR116 overexpression also lowered the elevation of APAP-induced ER stress marker protein levels (Fig. 5D). Correspondingly, IHC staining revealed that GPR116 overexpression weakened CHOP expression in liver tissues (Fig. 5E). To provide additional evidence for the protective role of GPR116, we administered ADV-*GPR116* to *GPR116*^△HC^ mice. Consistent with our expectations, the overexpression of GPR116 mitigated the hepatotoxicity induced by APAP in *GPR116*^△HC^ mice (Fig. 6A, B) and counteracted the elevated expression of CHOP resulting from GPR116 knockout (Fig. 6C, D). In summary, our findings establish GPR116 as a crucial hepatoprotective gene in the context of APAP-induced hepatotoxicity.

### GPR116 modulates ER stress in APAP-challenged mouse hepatocytes via β-arrestin1

**Fig. 7:**
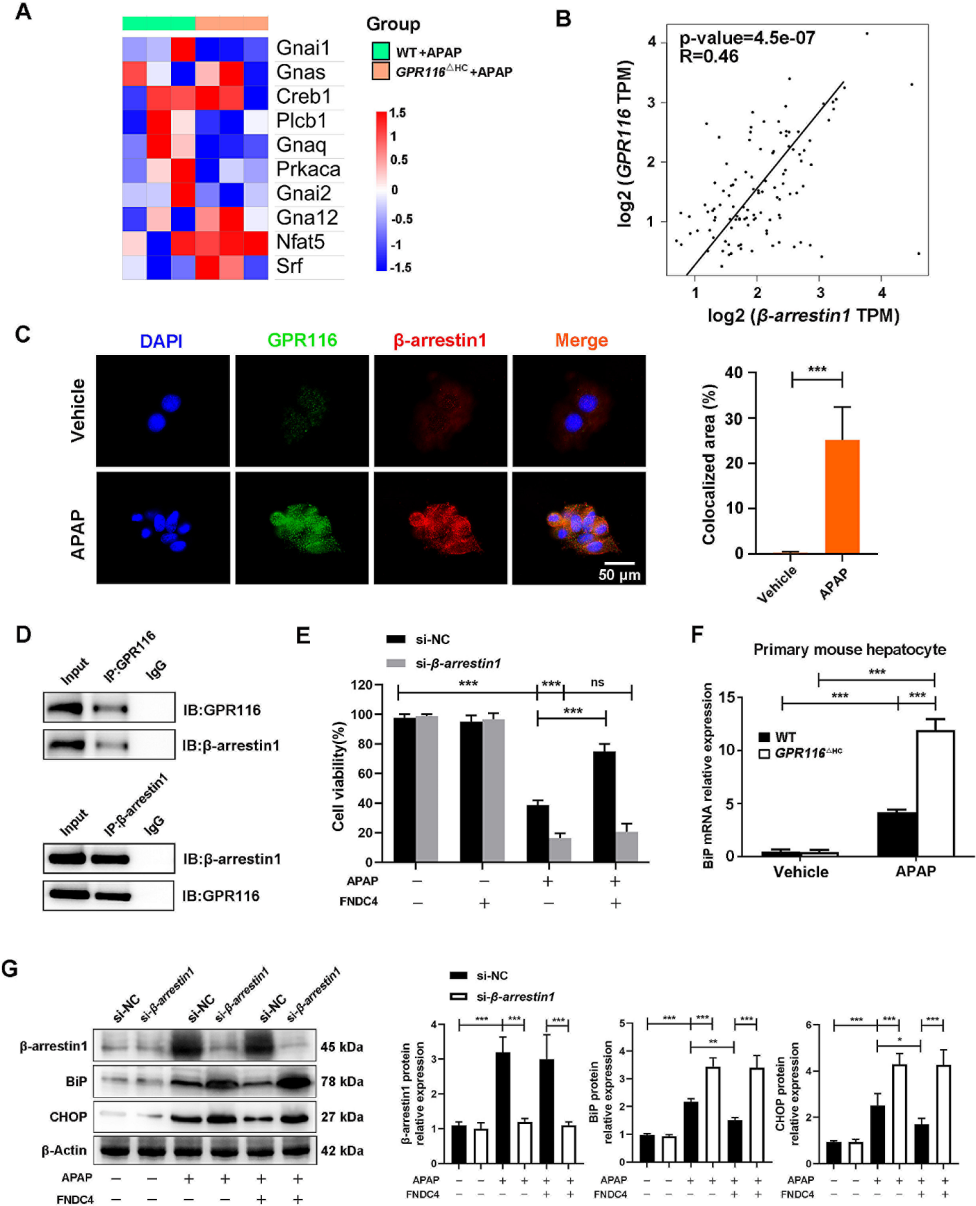
GPR116 inhibit ER stress in APAP-challenged mouse hepatocytes via β-arrestin1. (**A**) Heatmap of the differentially expressed genes in the RNA-seq data. (**B**) Co-expression analysis of GPR116 and β-arrestin1 in normal livers based on Q-PCR data. The value of each gene represents the relative expression normalized to HEK293T cells. Correlation coefficients (R) and *P* values were calculated by Spearman’s correlation analysis. (**C**) Representative double IF staining images and quantification showing the colocalization of GPR116 (green) and β-arrestin1 (red) in mouse primary hepatocytes treated with 10 mM APAP for 2 h (scale bar: 50 μm). (**D**) CO-IP assays showed that GPR116 interacted with β-arrestin1. Immunoprecipitations were performed by using anti-GPR116 (left panel) and anti-β-arrestin1 (right panel). (**E**) Primary mouse hepatocytes were transfected with si-NC or si-*β-arrestin1* for 24 h before and subjected to 30 min of pretreatment with FNDC4 (200 nM) or PBS before APAP treatment (10 mM, 2 h). Cell viability was assessed using CCK-8. (**F**) BiP mRNA expression in primary mouse hepatocytes treated with 10 mM APAP for 2 h. (**G**) Primary mouse hepatocytes were transfected with si-NC or si-*β-arrestin1* for 24 h before and subjected to 30 min of pretreatment with FNDC4 (200 nM) or PBS before APAP treatment (10 mM, 2 h). Protein levels of β-arrestin1, BiP and CHOP. Statistical analysis was performed by one-way ANOVA. Data are expressed as the mean ± SD. *P < 0.05; **P < 0.01; ***P < 0.001; ns *P* > 0.05 as indicated. Data are pooled from three independent experiments

We analyzed above RNA-seq data (Fig. 3) and found that knocking out *GPR116* did not affect the expression of classical G protein pathway-related genes during AILI (Fig. 7A), indicating that GPR116 may influence AILI through non-classical pathways. Recent research has revealed the role of the β-arrestin1 non-classical pathway in downstream signaling of GPR116 [29]. Therefore, we first analyzed the correlation between GPR116 and β-arrestin1 and found a positive correlation (*R* = 0.46) between the expression of *GPR116* and *β-arrestin1* mRNA expression in human normal liver tissue sequencing data from the GTEx database (Fig. 7B). Subsequent double IF staining and CO-IP experiments confirmed an interaction between GPR116 and β-arrestin1 in primary mouse hepatocytes following APAP challenge (Fig. 7C, D). The roles of β-arrestin1 were determined in vitro using small-interfering RNA (siRNA), si-*β-arrestin1*, to knock down the expression of β-arrestin1 in GPR116 signaling in mouse hepatocytes, and hepatocyte toxicity of APAP was detected using cell counting kit-8 (CCK-8) assay. si-*β-arrestin1* administration substantially diminished the protective effect induced by FNDC4, a ligand of GPR116, during APAP challenge (Fig. 7E), suggesting that GPR116 activation protects hepatocytes from APAP toxicity through β-arrestin1.

Studies have highlighted the inhibitory role of β-arrestin1 in ER stress, which may be achieved through negative regulation of BiP by β-arrestin1 [30]. BiP, a member of the heat shock protein 70 (Hsp70) family located in the ER, plays a crucial role in regulating ER functions [31]. Upon ER stress, BiP associates with unfolded or misfolded proteins in the ER, leading to the activation of PERK and IRE1α and ATF6 [32]. GO enrichment analysis revealed that GPR116 significantly affected chaperone/heat shock protein-related protein folding after APAP challenge (Fig. 3C, red arrow). Besides, we found that the level of *BiP* mRNA in APAP-stimulated *GPR116*^△HC^ primary hepatocytes was significantly higher than that in WT mice (Fig. 7F). In contrast, in primary hepatocytes obtained from WT mice, si-*β-arrestin1* reversed the FNDC4-induced decline in BiP and CHOP expression during the APAP challenge (Fig. 7G). In summary, GPR116 activation inhibits ER stress through the β-arrestin1-BiP signaling pathway in the APAP challenge.

### The therapeutic role of GPR116 activation in APAP-induced hepatotoxicity in mice

**Fig. 8:**
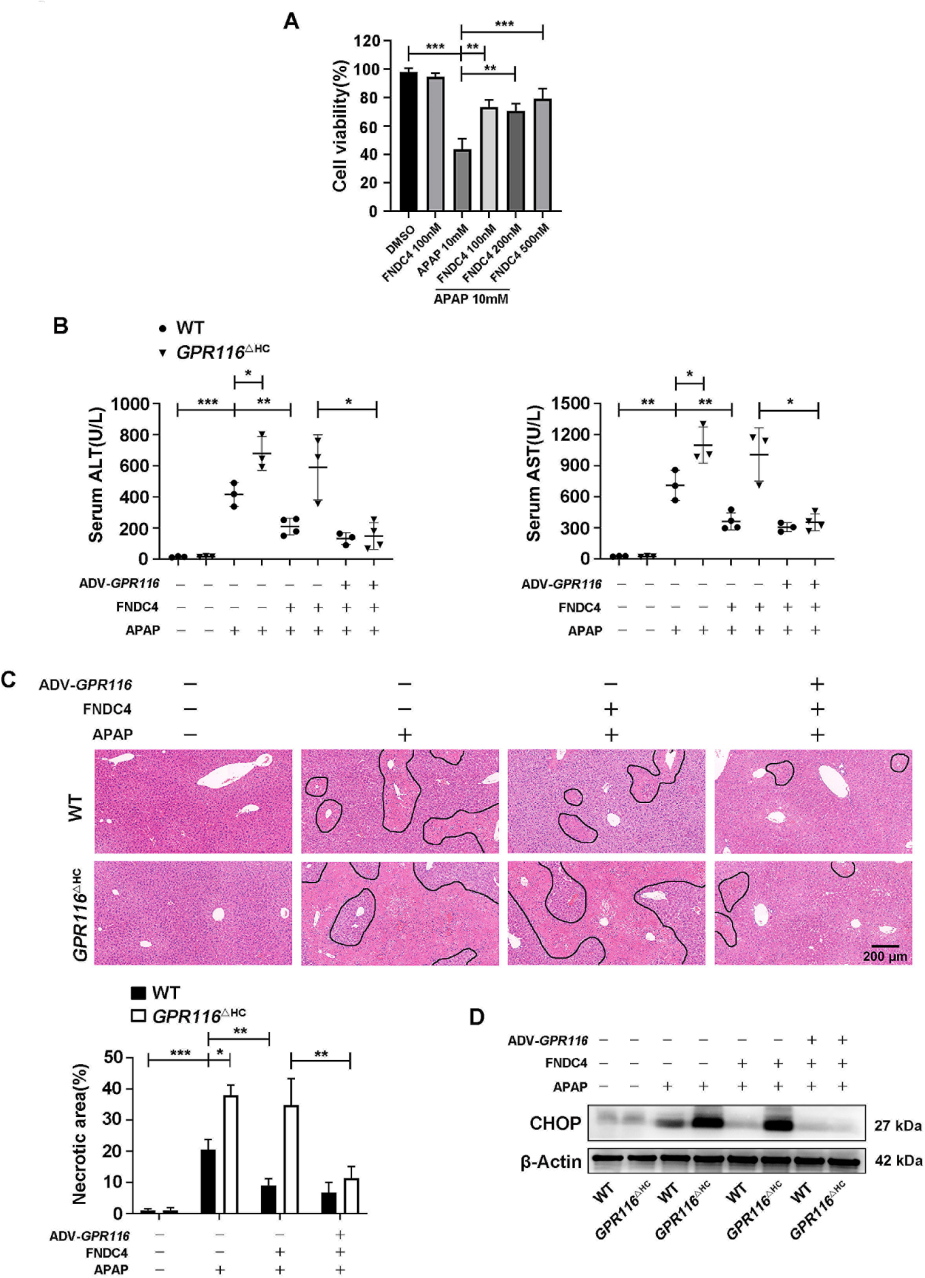
The therapeutic role of GPR116 activation in APAP-induced hepatotoxicity in mice. (**A**) Primary mouse hepatocytes were treated with FNDC4 (100/200/500 nM) or PBS 30 min after stimulated with APAP (10 mM). Cell viability was assessed using CCK-8 after 2 h (*n* = 3 independent experiments). (**B**-**D**) WT and *GPR116*^△HC^ mice were injected with ADV-RAM or ADV-*GPR116* 5 days before and subjected to FNDC4 (0.2 mg/kg, IP) or vehicle (PBS) 1 h after APAP treatment (250 mg/kg, IP) (*n* = 3–4 per group). (**B**) Serum levels of ALT and AST in mice 4 h after APAP injection. (**C**) Representative H&E staining of liver tissues from mice (scale bar: 200 μm) and quantification of necrotic areas 4 h after APAP injection (*n* = 3 per group). (**D**) Western blotting of CHOP in liver tissue homogenates of mice 4 h after APAP injection. Statistical analysis was performed by one-way ANOVA. Data are expressed as the mean ± SD. *P < 0.05; **P < 0.01; ***P < 0.001 as indicated. Data are pooled from at least two independent experiments

Considering the clinical significance of therapeutic administration in APAP overdose, previous reported synthetic peptides GAP16 and FNDC4 that activate GPR116 were administered in AILI. Notably, GAP16 did not alleviate hepatocellular toxicity of APAP in vitro or in vivo (Fig. S6A-C). Remarkably, our findings indicated that recombinant FNDC4 effectively alleviated APAP-induced hepatocellular toxicity in vitro (Fig. 8A). To investigate whether FNDC4 can alleviate AILI by activating GPR116 in vivo, we used ADV to overexpress GPR116 in *GPR116*^△HC^ mice. Interestingly, FNDC4 reduced AILI in WT mice, but did not improve liver damage in *GPR116*^△HC^ mice at 4 h post-APAP. More importantly, FNDC4 significantly alleviated liver injury in GPR116 re-expressing *GPR116*^△HC^ mice (Fig. 8B, C). Consistent with these findings, FNDC4 attenuated the upregulation of CHOP protein levels in WT and GPR116 re-expressing *GPR116*^△HC^ mice (Fig. 8D). However, 12 to 24 h post-APAP, FNDC4 administration failed to show protection in WT-APAP mice (Fig. S7A). The collective administration of GPR116 stimulation demonstrated protective effects against APAP in mice. Therefore, GPR116 holds promise as a valuable therapeutic approach for the management of AILI.

## Discussion

The aim of this research was to examine the intrinsic protective mechanisms of AILI and lay a rational groundwork for the advancement of novel therapeutic strategies. This investigation represents the initial documentation of the protective role of GPR116 in AILI progression. The upregulation of GPR116 in the liver was observed in both human subjects and murine models with AILI. The targeted deletion of *GPR116* in hepatocytes notably exacerbated APAP-induced liver injury and mortality in mice. Mechanistically, GPR116 protected hepatocytes from APAP-induced toxicity by suppressing ER stress via the β-arrestin1-BiP signaling pathway.

The expression of GPR116 was increased by APAP in the early stages. Given the acute nature of APAP overdose and the observed effects of GPR116 activation in WT-APAP mice, it is possible that APAP-induced GPR116 expression serves as a protective feedback mechanism. GPR116 owns a variety of important physiological homeostasis maintenance effects [17, 21, 22, 29], which is consistent with our results. Prior research has demonstrated that GPR116 in alveolar macrophages and neutrophils has the ability to suppress exaggerated inflammatory reactions [18, 33]. However, our IF staining revealed a notable enrichment of GPR116 in hepatocytes compared to non-parenchymal cells. These findings serve as a compelling rationale for further investigation into the role of GPR116 in hepatocytes.

During AILI, the main target of NAPQI is mitochondrial proteins prompting mechanism studies on AILI to focus on mitochondrial oxidative stress and dysfunction [34]. N-acetylcysteine (NAC) has been recommended by the FDA as the only treatment option for patients with APAP overdose; however, the application of this drug is very limited due to its side effects and narrow treatment window [35]. Thus, there is a need to explore possible treatments for other cellular events. Various hepatic pathologies, including drug toxicity, have been established to be linked to ER stress [36]. Usually, activation of UPR is sufficient to handle transient and mild forms of ER stress [37]. Nonetheless, if ER stress is severe and unresolved, it can lead to persistent activation of the UPR signaling pathway, resulting in cell death and accelerated organ damage. ER stress plays a key role in the early stage of hepatotoxicity of APAP, making it crucial to study the mechanism of UPR activation and homeostasis maintenance during AILI. The activation of GPCRs expressed in various cell types has been found to regulate ER stress [38], although the relationship between GPR116 and ER stress has not been previously explored. Following stimulation with tunicamycin, the expression of GPR116 in primary mouse hepatocytes remained unaltered (Fig. S5C), indicating that GPR116 is upstream of ER stress. Our research discovered, for the first time, the negative regulatory effect of GPR116 on ER stress. This finding demonstrates the critical role of GPR116 in maintaining ER homeostasis and provides a theoretical basis for further exploring the role of GPR116 in other liver diseases.

Following stimulation with APAP, we noted an increase in β-arrestin1 expression in hepatocytes, which co-localized with the upregulated GPR116. The inhibition of ER stress by GPR116 was found to be dependent on β-arrestin1. β-arrestin1, a widely expressed intracellular regulator of GPCR trafficking and desensitization, has been recognized as an independent mediator of GPCR signaling [39]. Through interactions with various signaling proteins, β-arrestin1 facilitates the formation of signaling protein complexes and the activation of downstream kinase cascades [40]. It has been reported that β-arrestin1 can inhibit ER stress in intestinal stem cell proliferation induced by radiation [41]. The role of BiP as a crucial regulator of ER stress makes it a potential therapeutic target in various physiological and pathological processes [42, 43]. Particularly, precision treatment approaches that focus on specific disease states or cell types can benefit from the therapeutic potential of BiP [44]. Our findings supported that targeting the GPR116-β-arrestin1-BiP may present a novel therapeutic strategy for the management of AILI. CHOP is a key regulator of AILI. During AILI, CHOP upregulation may compromise hepatocyte survival via various mechanisms [7]. Accordingly, inhibiting CHOP could mitigate further injury to the liver following APAP toxicity. Our study demonstrated that GPR116 inhibited CHOP expression enhancement, resulting in decreased hepatocyte necrosis.

GPR116, an aGPCR, features a conserved GPS. Previous studies have demonstrated receptor activation by its corresponding tethered agonist GAP16 in vitro [45]. However, despite these findings, GAP16 failed to alleviate hepatocyte toxicity of APAP both in vivo and in vitro, which was unexpected. A functional analysis of the stachel sequences and derived peptides revealed agonist promiscuity both within and between aGPCR subfamilies [46]. Hence, potential functional overlap must be considered for in vitro and in vivo studies. Nevertheless, the functional antibody (anti-GPR116) targeting the extracellular part of GPR116 blocks a wider range of sites and effectively prevented the inhibitory effect of GPR116 on PERK-CHOP signal channel (Fig. S6E).

This study focused on investigating the role and mechanism of GPR116 during the early stage of AILI. The expression of GPR116 increased shortly after APAP stimulation, which is consistent with the occurrence of ER stress during the early phase of APAP-induced hepatotoxicity. In contrast, the mechanism of AILI during later stages is complex [47]. It involves processes such as inflammation, mitochondrial damage, and liver repair, which are not the focal point of this study. Our study found that GPR116 overexpression can alleviate the degree of liver injury in mice. However, the up-regulation of endogenous GPR116 expression in mice stimulated by APAP under physiological conditions did not significantly protect against liver injury. This is potentially due to that endogenous GPR116 expression was up-regulated by 2–3 times after APAP stimulation, while the ADV-induced GPR116 overexpression was up-regulated by up to 800 times. The significant difference in the effect caused by such a huge disparity in expression level was understandable.

FNDC4, a type of hepatokine, has been reported to exert anti-inflammatory effects on macrophages and osteoclasts, promoting survival in response to severe chronic inflammation [48, 49]. Furthermore, FNDC4 suppresses ER stress in adipocytes, which reduces insulin resistance caused by hyperlipidemia [50]. In white adipose tissue, GPR116 has been discovered to be a receptor for FNDC4. Upon direct binding of FNDC4 to GPR116, insulin signaling and insulin-mediated glucose uptake are promoted in white adipocytes [22]. Our study found that FNDC4 could not improve APAP-induced hepatocyte toxicity in mice lacking GPR116 specifically in hepatocytes, it did alleviate liver injury and ER stress to some extent in GPR116 re-expressing *GPR116*^△HC^ mice, supporting the notion that FNDC4 primarily targets hepatocyte GPR116. Notably, FNDC4 administration exhibited initial liver protection, however, its efficacy diminished between 12 and 24 h post-APAP injection. This observation aligns with the role of GPR116 as an early protective response in AILI. The intricate nature of AILI during later stages, characterized by inflammation, mitochondrial damage, and liver repair processes, may contribute to the waning protective effects of FNDC4.

Extensive necrosis in cases of APAP overdose leads to sterile inflammation and the subsequent recruitment of inflammatory cells [51]. While our findings indicate a lack of significant alteration in the expression of GPR116 in macrophages, neutrophils, and hepatic stellate cells, further investigation is needed to elucidate the role of GPR116 in APAP-induced inflammation. Recent advancements in technology have enabled the direct dynamic observation of mouse liver microenvironments [52, 53]. By introducing fluorescently labeled cells, real-time monitoring within the live mouse liver can track the migration and distribution of inflammatory cells [54]. This technique will be utilized in our future studies to gain a more comprehensive understanding of the effects of GPR116 on the mouse liver, including the distribution of inflammatory cells, the progression of hepatic pathologies, and the evaluation of therapeutic effectiveness.

## Conclusions

Our study has provided evidence in both mice and humans indicating that GPR116 serves as a crucial endogenous protective factor in AILI, protecting against hepatocyte necrosis and liver damage induced by excessive APAP through the inhibition of ER stress via the β-arrestin1-BiP signaling pathway. These findings have the potential to enhance our understanding of the mechanisms underlying AILI.

### Electronic supplementary material

Below is the link to the electronic supplementary material.

Supplementary Material 1

## Data Availability

The raw data that support the findings of this study are available from the corresponding author, Jinjun Bian upon reasonable request.

## Abbreviations

APAP: Acetaminophen
AILI: APAP-induced liver injury
aGPCRs: Adhesion GPCRs
GPR116: G protein-coupled receptor 116
ADV: Adenoviruses
ER: Endoplasmic reticulum
BiP: Binding immunoglobulin protein
FNDC4: Fibronectin type III domain containing 4
CYP2E1: Cytochrome P 450 enzymes 2E1
NAPQI: N-acetyl-p-benzoquinone imine
GSH: Glutathione
UPR: Unfolded protein response
IHC: Immunohistochemistry
IP: Intraperitoneally
TUDCA: Tauroursodeoxycholic acid
ALT: Alanine aminotransferase
AST: Aspartate aminotransferase
Q-PCR: Quantitative real time polymerase chain reaction
H&E: Hematoxylin and Eosin
TUNEL: TdT-mediated dUTP Nick-end labeling
IF: Immunofluorescence
SD: Standard deviation
MPO: Myeloperoxidase
α-SMA: Alpha-smooth muscle actin
WT: Wild type
APAP-CYS: APAP-cysteine protein adducts
KEGG: Kyoto encyclopedia of genes and genomes
GO: Gene ontology
Hsp70: Heat shock protein 70
CO-IP: Co-Immunoprecipitation
ATF4: Activating transcription factor 4
ATF6: Activating transcription factor 6
PDI: Protein disulfide isomerase
XBP-1: X-box binding protein-1
CHOP: C/EBP-homologous protein
eIF2α: Eukaryotic initiation factor 2α
IRE1α: Inositol-requiring enzyme 1α
PERK: Protein kinase R-like ER kinase
CCK-8: Cell counting kit-8
NAC: N-acetyl cysteine
GPS: GPCR proteolysis site
