# Supplementary material for: GPR116 alleviates acetaminophen-induced liver injury in mice by inhibiting endoplasmic reticulum stress
Source: Cell Mol Life Sci. 2024 Jul 13;81(1):299. doi: 10.1007/s00018-024-05313-0 (PMC11335223; doi:10.1007/s00018-024-05313-0)
Supplement: Supplementary file 1 — Supplementary Material 1 [file 18_2024_5313_MOESM1_ESM.docx]

**Supplementary material**

**GPR116 Alleviates Acetaminophen-induced Liver Injury in Mice by Inhibiting Endoplasmic Reticulum Stress**

Author: Qian Xiang ^a,b,1^, Na Li ^a,1^, Yan Zhang ^a,1^, Ting Wang^a^, Ying Wang^a^, Jinjun Bian^a,^*

^a^ Faculty of Anesthesiology, Changhai Hospital, Naval Medical University, Shanghai 200433, China

^b^ Department of Anesthesiology, Peking University Third Hospital, Beijing 100191, China.

^1^These authors contributed equally to this work

* Corresponding author

**Supplementary Methods**

**RNA sequencing and analysis**

Liver tissues harvested from GPR116^△HC^ and WT mice treated with APAP (250 mg/kg, IP) or an equal volume of saline (IP) as vehicle control for 4 h were subjected to RNA-seq analysis (n = 3 per group). Total RNA was extracted from the liver tissues using TRIzol® Reagent according the manufacturer’s instructions (Magen). RNA samples were detected based on the A260/A280 absorbance ratio with a Nanodrop ND-2000 system (Thermo Scientific, USA), and the RIN of RNA was determined by an Agilent Bioanalyzer 4150 system (Agilent Technologies, CA, USA). Only qualified samples will be used for library construction.

Paired-end libraries were prepared using an ABclonal mRNA-seq Lib Prep Kit (ABclonal, China) following the manufacturer’s instructions. The mRNA was purified from 1 μg total RNA using oligo (dT) magnetic beads followed by fragmentation carried out using divalent cations at elevated temperatures in ABclonal First Strand Synthesis Reaction Buffer. Subsequently, first-strand cDNAs were synthesized with random hexamer primers and reverse transcriptase (RNase H) using mRNA fragments as templates, followed by second-strand cDNA synthesis using DNA polymerase I, RNAseH, buffer, and dNTPs. The synthesized double stranded cDNA fragments were then adapter ligated for preparation of the paired-end library. Adaptor-ligated cDNA was used for PCR amplification. PCR products were purified (AMPure XP system), and library quality was assessed on an Agilent Bioanalyzer 4 150 system. Finally, the library preparations were sequenced on an Illumina Novaseq 6 000 (or MGISEQ-T7) and 150 bp paired-end reads were generated. GO analysis was performed to elucidate the biological implications of unique genes in the significant or representative profiles of the target gene of the differentially expressed mRNA in the experiment. Pathway analysis was used to determine the significant pathways of the differentially expressed genes according to the KEGG database.

**Supplementary Figures**


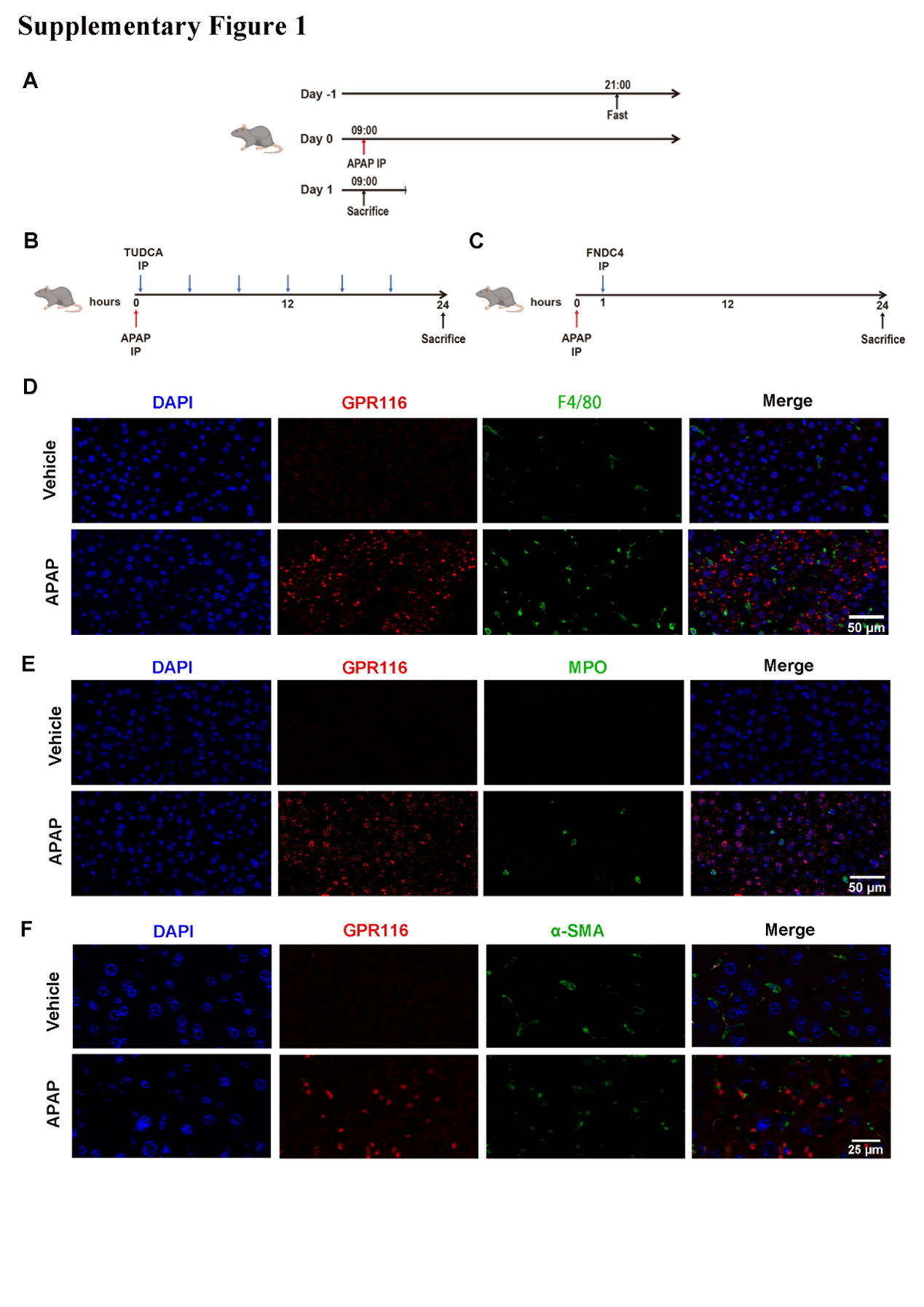


**Fig. S1.**

(A) Mice fasted overnight were injected with APAP (250 mg/kg, IP) and the livers were harvested post 24h. (B) Mice were injected with APAP (250 mg/kg, IP) with or without TUDCA treatment (250 mg/kg, IP, 3 times/12 h) and the livers were harvested post 24h. (C) Mice were injected with APAP (250 mg/kg, IP) with or without FNDC4 treatment (0.2 mg/kg, IP, 1 h after APAP injection) and the livers were harvested post 24h. (D) Representative double IF staining images showing GPR116 (red) and F4/80 (green) in liver tissues of mice treated with APAP for 4 h (scale bar: 50 μm). (E) Representative double IF staining images showing GPR116 (red) and MPO (green) in liver tissues of mice treated with APAP for 4 h (scale bar: 50 μm). (F) Representative double IF staining images showing GPR116 (red) and α-SMA (green) in liver tissues of mice treated with APAP for 4 h (scale bar: 25 μm).


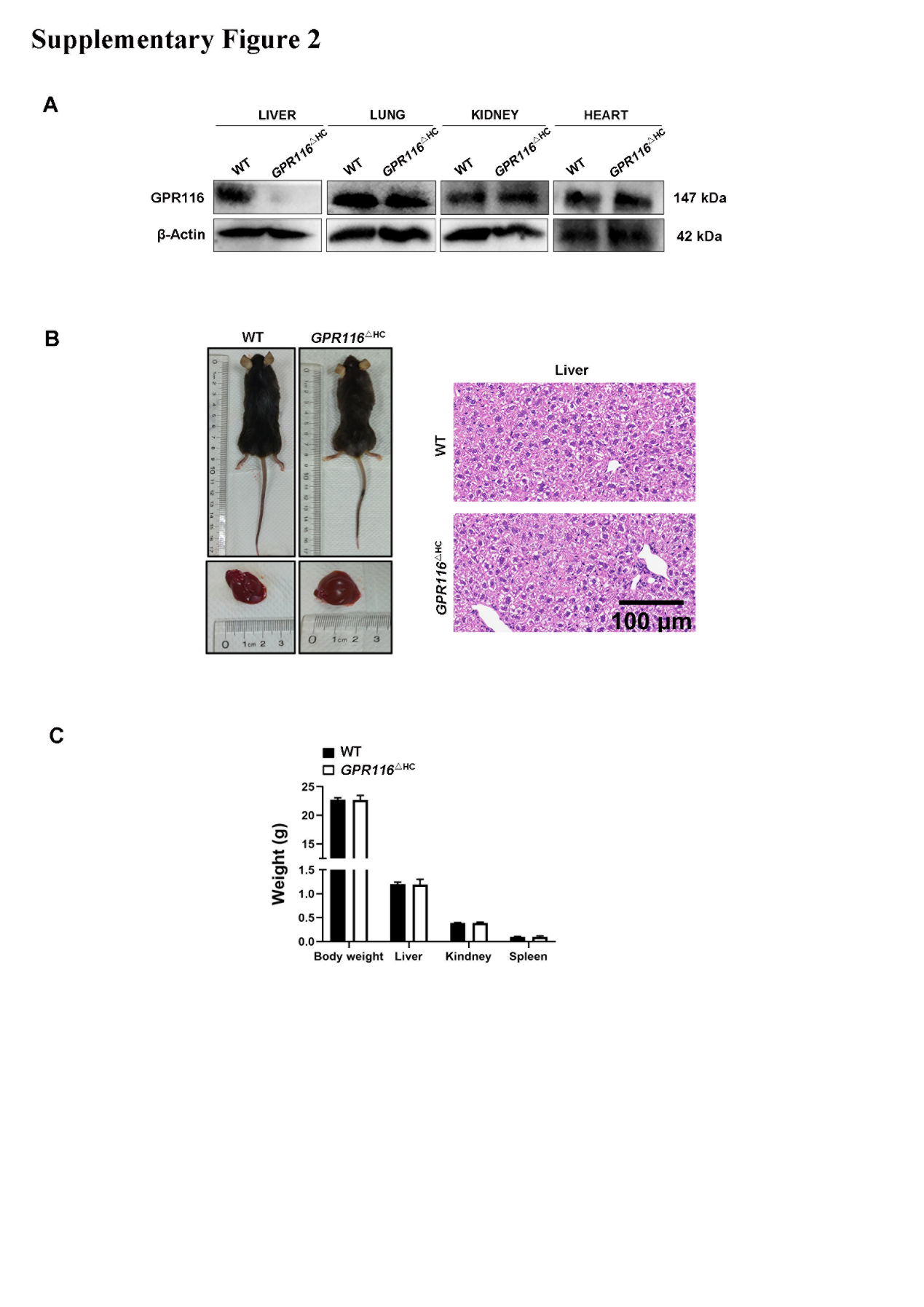


**Fig. S2.**

(A) Protein levels of GPR116 in the liver, lung, kidney, and heart of GPR116^Hep+/+^ (WT) and *GPR116*^△HC^ mice were determined by western blotting. (B) Unchallenged 8-week-old male WT and *GPR116*^△HC^ mice. Shown are representative gross appearance of mice and livers, H&E staining (scale bar: 100 μm) of liver tissues. (C) Body, liver, kidney, and spleen tissue weights. Statistical analysis was performed by one-way ANOVA. Data are expressed as the mean ± SD. **P* <0.05; ***P* <0.01; ****P* <0.001; ns *P* >0.05 as indicated.


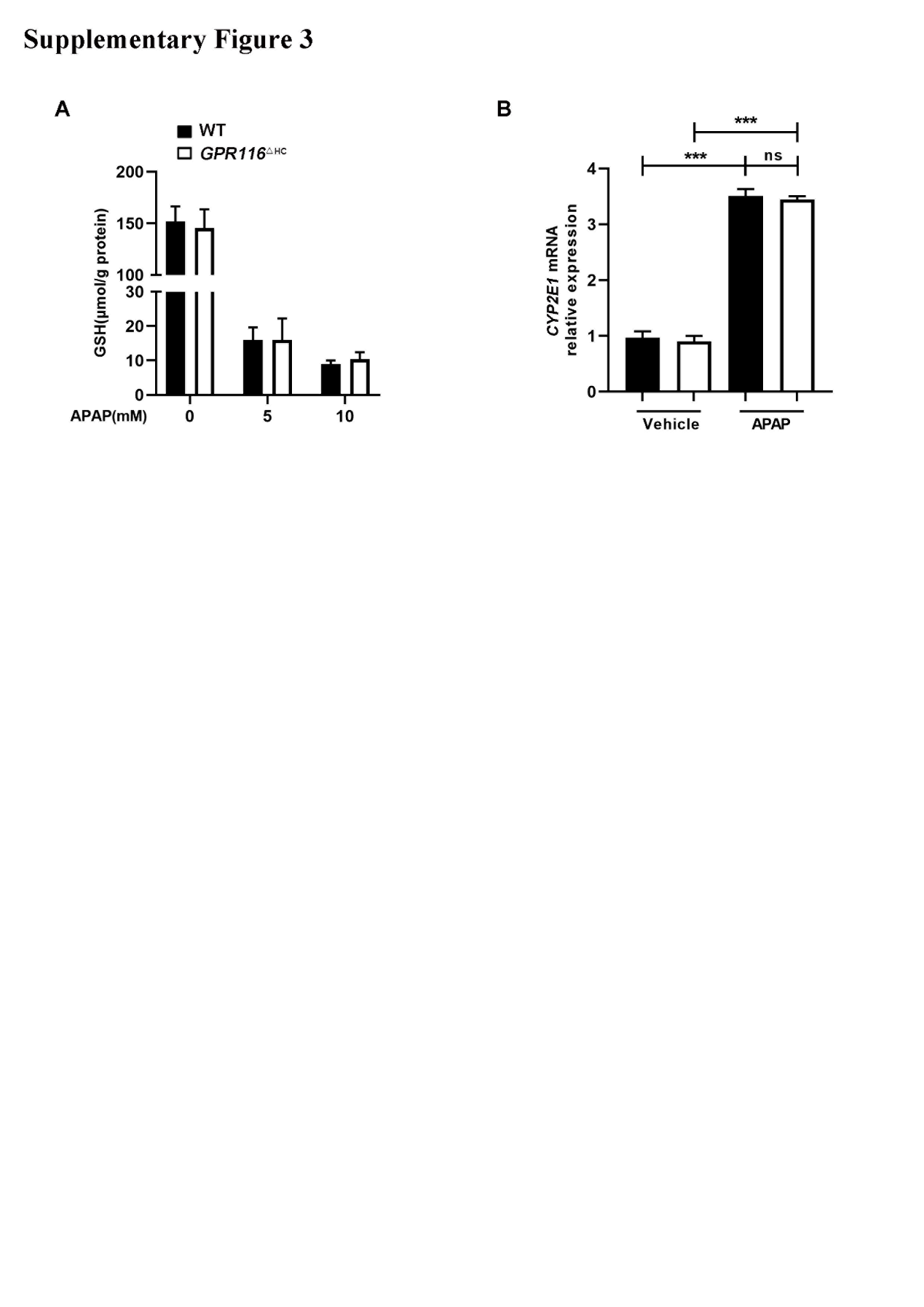


**Fig. S3.**

(A) GSH level in primary mouse hepatocytes treated with 5/10 mM APAP for 2h (n = 3 independent experiments). (B) *CYP2E1* mRNA expression in primary mouse hepatocytes treated with 10 mM APAP for 2h (n = 3 independent experiments). Statistical analysis was performed by one-way ANOVA. Data are expressed as the mean ± SD. **P* <0.05; ***P* <0.01; ****P* <0.001; ns *P* >0.05 as indicated.


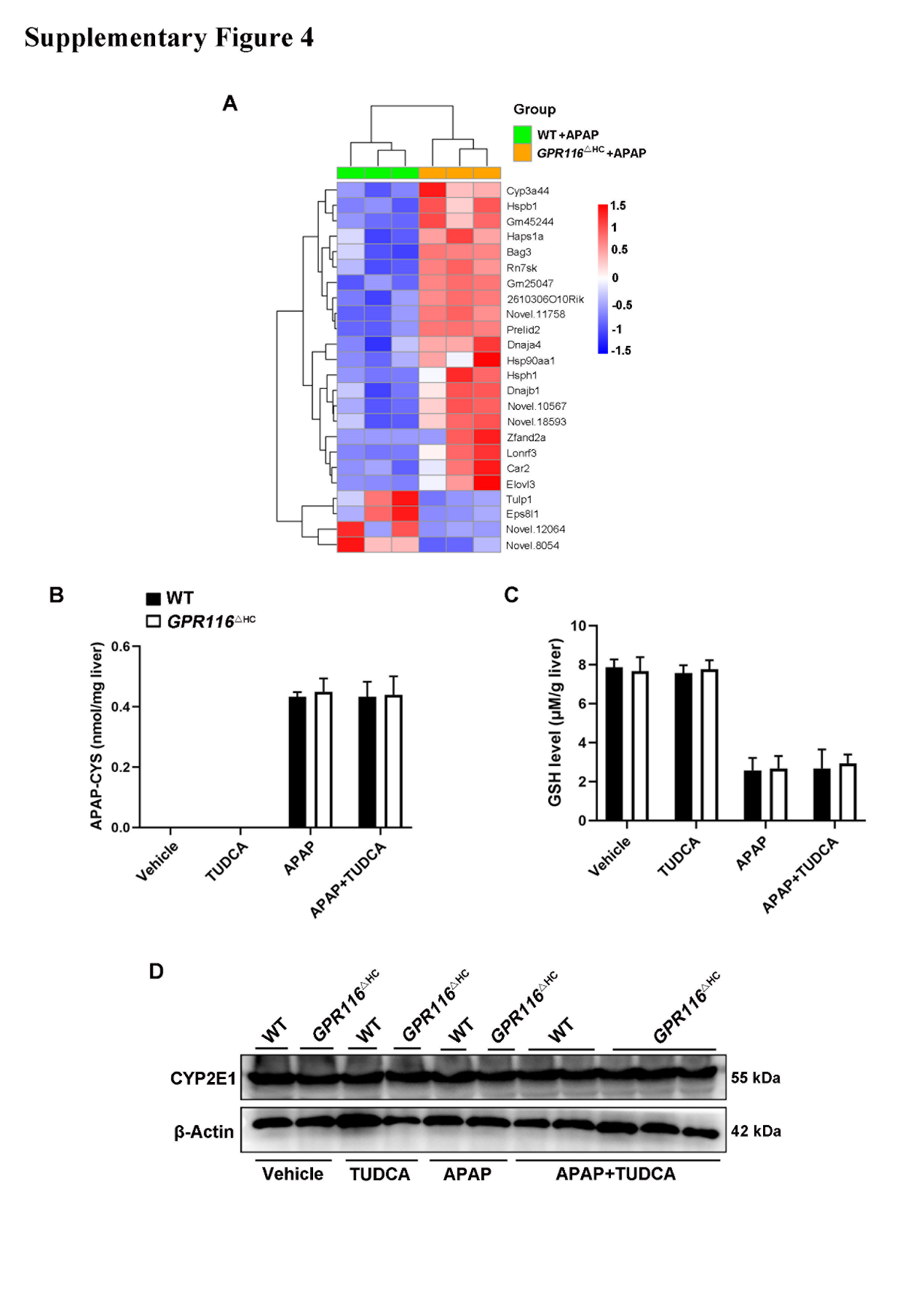


**Fig. S4.**

WT and *GPR116*^△HC^ mice were injected with either APAP (250 mg/kg, IP) or an equal volume of saline (IP) as a vehicle control for 4 h and liver tissues for RNA‐seq analysis (n = 3 per group). (A) Heatmap of the differentially expressed genes in the RNA-seq data. (B-D) The APAP-CYS level, GSH level and CYP2E1 protein level in the liver tissue homogenates of mice. Statistical analysis was performed by one-way ANOVA. Data are expressed as the mean ± SD. **P* <0.05; ***P* <0.01; ****P* <0.001; ns *P* >0.05 as indicated. Data (C) are pooled from at least two independent experiments.


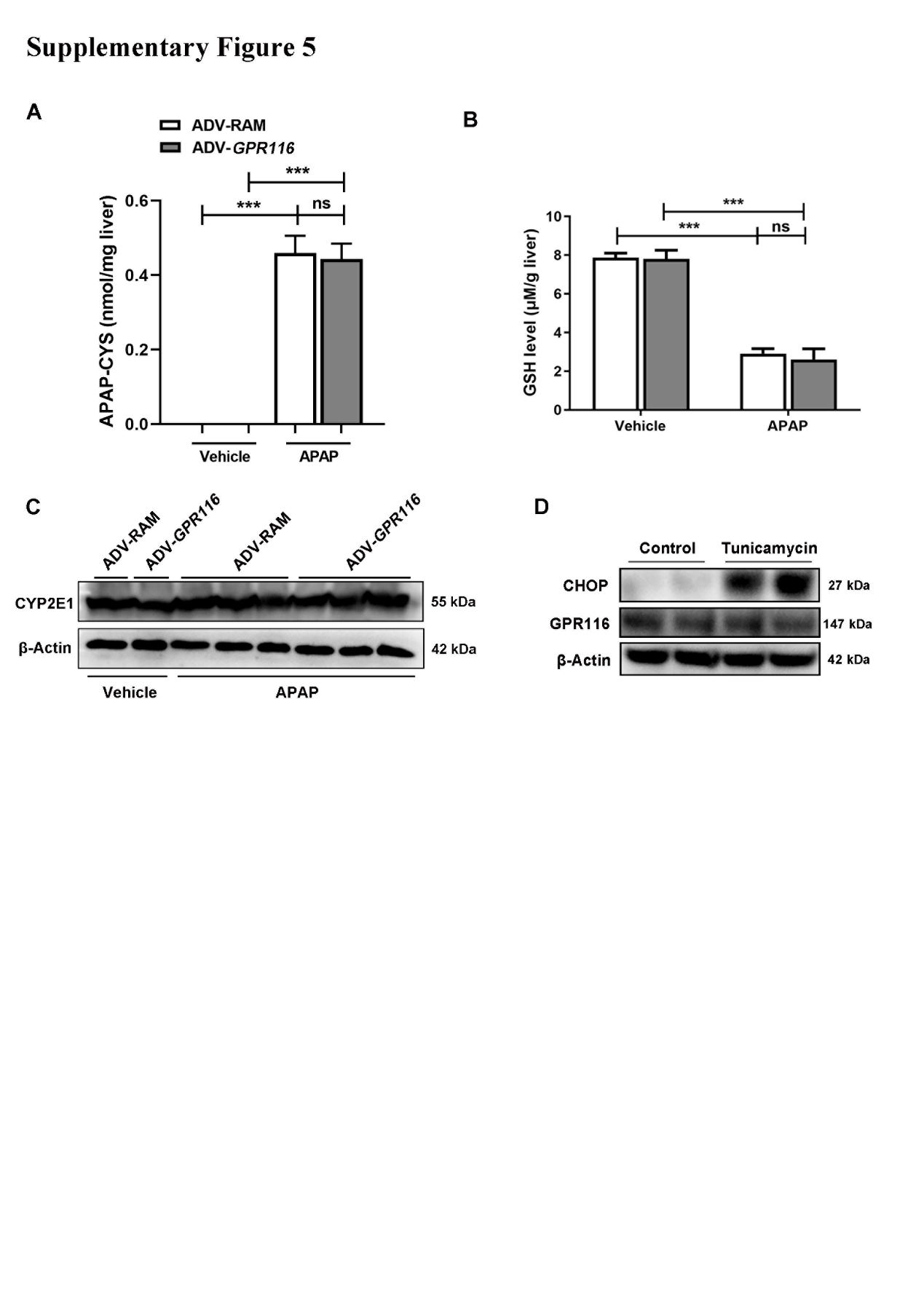


**Fig. S5.**

(A-C) The APAP-CYS level, GSH level and CYP2E1 protein level in the liver tissue homogenates of mice 4 h after APAP injection. (D) Protein levels of CHOP and GPR116 in mouse primary hepatocytes treated with 10 μg/ml tunicamycin for 6 h (n = 3 independent experiments). Statistical analysis was performed by one-way ANOVA. Data are expressed as the mean ± SD. **P* <0.05; ***P* <0.01; ****P* <0.001; ns *P* >0.05 as indicated. Data are pooled from at least two independent experiments.


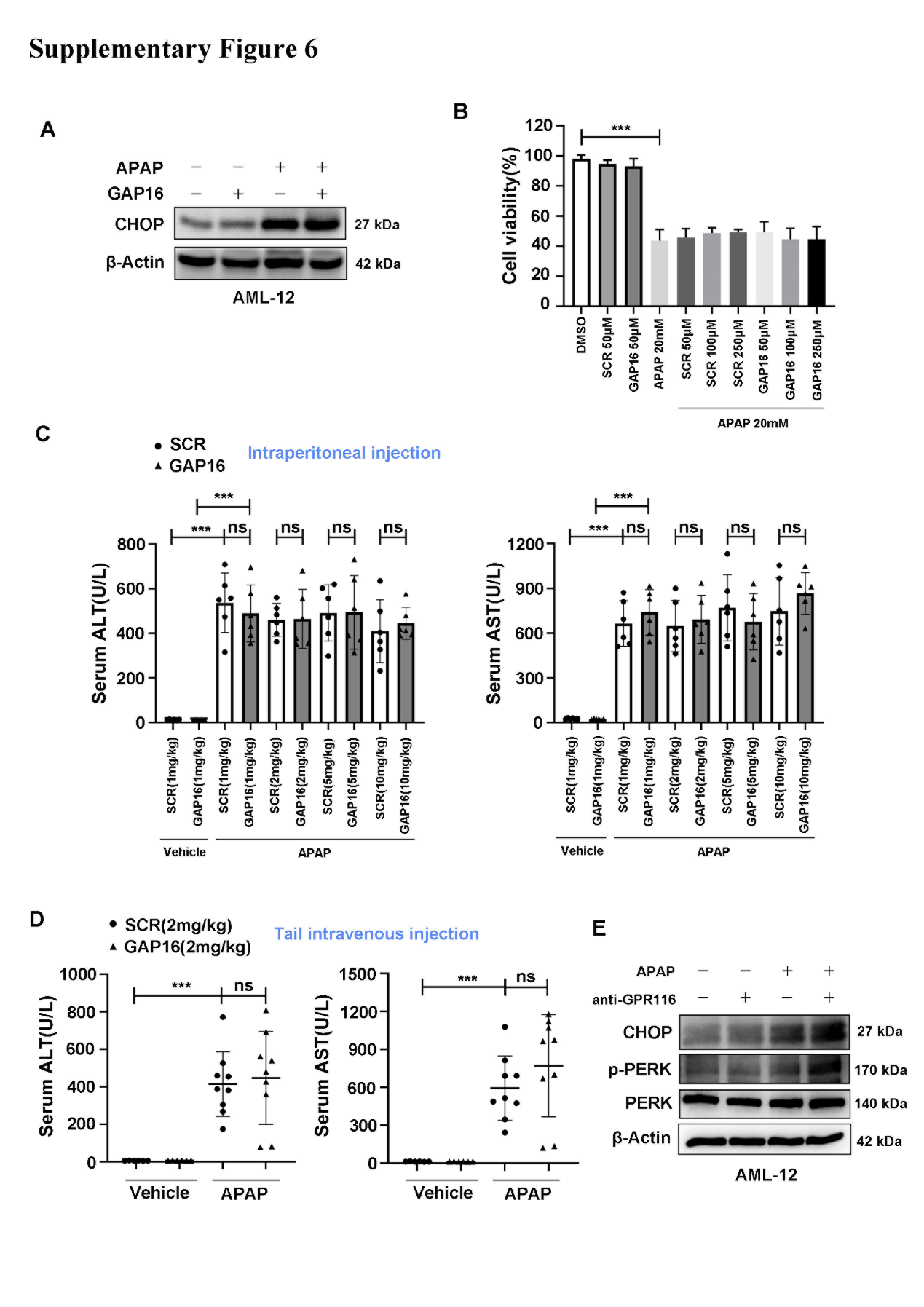


**Fig. S6.**

(A) Primary mouse hepatocytes were pretreated with GAP16 (250 μM) or SCR (vehicle control, 250 μM) for 30 min and then stimulated with APAP (10 mM) for 2 h. Western blotting of CHOP in cell lysates (n = 3 independent experiments). (B) Primary mouse hepatocytes were pretreated with GAP16 (50/100/250 μM) or SCR (vehicle control, 50/100/250 μM) for 30 min and then stimulated with APAP (10 mM) for 2 h. Cell viability was assessed using CCK-8 (n = 3 independent experiments). (C) C57BL/6J mice were pretreated with GAP16 or SCR (1/2/5/10 mg/kg, IP) for 1 h and then stimulated with APAP (250 mg/kg, IP) for 4 h (n = 6 per group). Serum levels of ALT and AST in mice. (D) C57BL/6J mice were pretreated with GAP16 or SCR (2 mg/kg, tail intravenous injection) for 1 h and then stimulated with APAP (250 mg/kg, IP) for 4 h (n = 6-9 per group). Serum levels of ALT and AST in mice. (E) Primary mouse hepatocytes were pretreated with anti-GPR116 (0.4 µg/ml) or isotype control (0.4 µg/ml) for 30 min and then stimulated with APAP (10 mM) for 2 h. Western blotting of CHOP and phospho-PERK/PERK in cell lysates (n = 3 independent experiments). Statistical analysis was performed by one-way ANOVA. Data are expressed as the mean ± SD. **P* <0.05; ***P* <0.01; ****P* <0.001 as indicated. Data are pooled from at least two independent experiments.


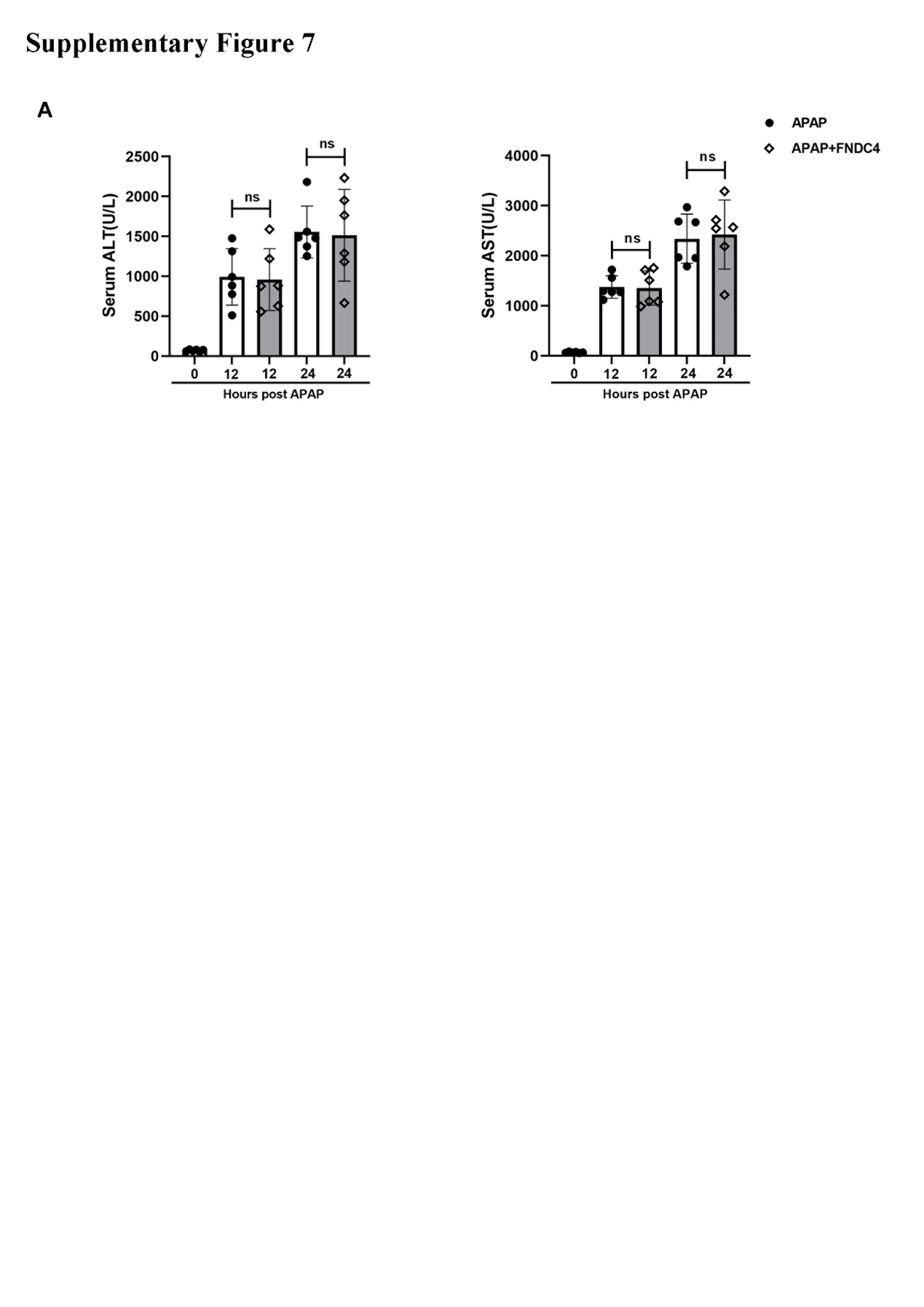


**Fig. S7.**

WT mice were subjected to FNDC4 (0.2 mg/kg, IP) or vehicle (PBS) 1 h after APAP treatment (250 mg/kg, IP) (n = 3-4 per group). (A) Serum levels of ALT and AST in mice 12 h and 24 h after APAP injection. Statistical analysis was performed by one-way ANOVA. Data are expressed as the mean ± SD. **P* <0.05; ***P* <0.01; ****P* <0.001; ns *P* >0.05 as indicated.
